# Development and extensive sequencing of a broadly-consented Genome in a Bottle matched tumor-normal pair for somatic benchmarks

**DOI:** 10.1101/2024.09.18.613544

**Published:** 2024-09-22

**Authors:** Jennifer H. McDaniel, Vaidehi Patel, Nathan D. Olson, Hua-Jun He, Zhiyong He, Anthony Schmitt, Kristin Sikkink, Fritz J Sedlazeck, Harsha Doddapaneni, Shalini N. Jhangiani, Donna M. Muzny, Marie-Claude Gingras, Heer Mehta, Luis F Paulin, Alex R Hastie, Hung-Chun Yu, Victor Weigman, Alison Rojas, Katie Kennedy, Jamie Remington, Isai Gonzalez, Mitch Sudkamp, Kelly Wiseman, Bryan Lajoie, Shawn Levy, Miten Jain, Stuart Akeson, Giuseppe Narzisi, Zoe Steinsnyder, Catherine Reeves, Jennifer Shelton, Sarah B. Kingan, Christine Lambert, Primo Bayabyan, Aaron M. Wenger, Ian J. McLaughlin, Aaron Adamson, Christopher Kingsley, Melanie Wescott, Young Kim, Benedict Paten, Jimin Park, Ivo Violich, Karen H Miga, Joshua Gardner, Brandy McNulty, Gail Rosen, Rajiv McCoy, Lesley Chapman Hannah, Doron Lipson, Hila Benjamin, Nika Iremadze, Ilya Soifer, Stephen Eacker, Mary Wood, Erin Cross, Greg Husar, Stephen Gross, Michael Vernich, Mikhail Kolmogorov, Tanveer Ahmad, Ayse Keskus, Asher Bryant, Francoise Thibaud-Nissen, Jonathan Trow, Justin Wagner, Chunlin Xiao, Andrew S Liss, Justin M. Zook, the Genome in a Bottle Consortium

**Affiliations:** 1. Material Measurement Laboratory, National Institute of Standards and Technology, 100 Bureau Dr., Gaithersburg, MD 20899, USA; 2. Arima Genomics, Inc., 6354 Corte Del Abeto Suite B, Carlsbad, CA 92011, USA; 3. Human Genome Sequencing Center, Baylor College of Medicine, Houston, TX, USA; Department of Molecular and Human Genetics, Baylor College of Medicine, TX, USA; Department of Computer Science, Rice University, 6100 Main Street, Houston, TX, USA; 4. Human Genome Sequencing Center, Baylor College of Medicine, OneBaylor Plaza, Houston, Texas 77030, USA; 5. Bionano Genomics; 6. BioSkryb Genomics, 2810 Meridian Pkwy, ste110, Durham, NC 27713; 7. Applications Development Laboratory, Element Biosciences, 10055 Barnes Canyon Rd, San Diego, CA 92121, USA; 8. Genome Technology Laboratory, Northeastern University, 360 Huntington Ave, Boston, MA 02115, USA; 9. New York Genome Center, New York, NY 10013, USA; 10. PacBio, 1305 O’Brien Drive, Menlo Park, CA 94025, USA; 11. UC Santa Cruz Genomics Institute, University of California, Santa Cruz, CA, USA; 12. Ecological and Evolutionary Signal-processing and Informatics (EESI) Lab, College of Engineering, Drexel University, Philadelphia, PA, USA; 13. Department of Biology, Johns Hopkins University, 3400 N. Charles St., Baltimore, MD 21218, USA; 14. National Cancer Institute, Division of Cancer Epidemiology and Genetics, Clinical Genetics Branch, National Institutes of Health, 9609 Medical Center Dr, Rockville, MD 20850, USA; 15. Ultima Genomics Inc., 4425 Technology Dr, Fremont, CA 94538, USA; 16. Phase Genomics, Inc. Seattle, WA, USA; 17. KromaTiD, 1880 Industrial Circle, Suite A, Longmont, Colorado 80501, USA; 18. Cancer Data Science Laboratory, National Cancer Institute, National Institutes of Health, 9000 Rockville Pike, Bethesda, MD 20892, USA; 19. National Center for Biotechnology Information, National Library of Medicine, National Institutes of Health, 45 Center Drive, Bethesda, MD, 20894, USA; 20. Department of Surgery, Massachusetts General Hospital and Harvard Medical School, Boston MA 02114, USA

## Abstract

The Genome in a Bottle Consortium (GIAB), hosted by the National Institute of Standards and Technology (NIST), is developing new matched tumor-normal samples, the first to be explicitly consented for public dissemination of genomic data and cell lines. Here, we describe a comprehensive genomic dataset from the first individual, HG008, including DNA from an adherent, epithelial-like pancreatic ductal adenocarcinoma (PDAC) tumor cell line (HG008-T) and matched normal cells from duodenal tissue (HG008-N-D) and pancreatic tissue (HG008-N-P). The data come from thirteen whole genome measurement technologies: Illumina paired-end, Element standard and long insert, Ultima UG100, PacBio (HiFi and Onso), Oxford Nanopore (standard and ultra-long), Bionano Optical Mapping, Arima and Phase Genomics Hi-C, G-banded karyotyping, directional genomic hybridization, and BioSkryb Genomics single-cell ResolveDNA. Most tumor data is from a large homogenous batch of non-viable cells after 23 passages of the primary tumor cells, along with some data from different passages to enable an initial understanding of genomic instability. These data will be used by the GIAB Consortium to develop matched tumor-normal benchmarks for somatic variant detection. In addition, extensive data from two different normal tissues from the same individual can enable understanding of mosaicism. Long reads also contain methylation tags for epigenetic analyses. We expect these data to facilitate innovation for whole genome measurement technologies, *de novo* assembly of tumor and normal genomes, and bioinformatic tools to identify small and structural somatic mutations. This first-of-its-kind broadly consented open-access resource will facilitate further understanding of sequencing methods used for cancer biology.

## Background & Summary

The GIAB Consortium has previously developed extensive sequencing data and benchmarks for seven normal human cell lines, which are used in technology development and validation of clinical assays.^1,2^ While some tumor cell lines have been characterized as benchmarks,^3–6^ these data are from legacy cell lines with no consent or consent before whole genome sequencing was routine. In 2013, NIH determined that researchers could publicly share genomic data from legacy cancer cell lines in the Cancer Cell Line Encyclopedia despite having no or limited consent, because most of these cell lines already had public genomic data available (https://www.cancer.gov/ccg/research/structural-genomics/tcga/history/policies/ccle-open-release-justification.pdf). However, the policy states that it could be revised in the future, and it “strongly recommended that informed consent processes for prospective research development of human cell lines (for research or commercial purposes) fully describe and consider any risks associated with broad distribution of genomic data derived from those cell lines.” Therefore, there is a need for new tumor-normal pairs with explicit consent for public dissemination of genome sequencing data and cell lines, so that they can be developed into enduring reference samples for benchmarking somatic variants.^7^

To meet this need, we describe a comprehensive dataset of whole genome-scale measurements, including extensive short and long-read sequencing, from a new immortal PDAC cell line and paired normal pancreatic and duodenal tissues, derived from a female of European genetic ancestry with explicit consent for public release of genomic data. We will make additional data and benchmarks available as they are generated. We are also actively working to deposit the cell line in a public repository. Links to these resources will be posted on the NIST Cancer Genome in a Bottle webpage: https://www.nist.gov/programs-projects/cancer-genome-bottle.

## Methods

### Pancreatic cancer patient with explicit consent for genomic data sharing

The GIAB Consortium requires explicit consent for genomic data sharing for its widely-used cell lines, NIST is collaborating with the Liss Laboratory at Massachusetts General Hospital (MGH) (https://www.massgeneral.org/surgery/gastrointestinal-and-oncologic-surgery/research/liss-laboratory) to develop matched tumor-normal cell lines. The Liss Lab operates the Pancreatic Tumor Bank at MGH. MGH recruited participants, performing tissue collection and developing matched tumor-normal cell lines from the resected tissues. The participants have been explicitly consented for tissue collection and public genomic data sharing following an MGH IRB-approved protocol (see relevant excerpt from the consent below). The MGH Data and Tissue Sharing Committee and NIST Research Protections Office determined that the signed consent allows for sharing of the participants’ genomic data and development and distribution of cell lines as reference materials.

#### Consent excerpt for sharing genomic data and cell line

For transparency, we include the parts of the MGH IRB-approved consent relevant to public sharing of genomic data, creation of the immortalized cell line, and public sharing of the cell line below:

“We plan to do genetic research on the DNA in your tissue sample. DNA is the material that makes up your genes. All living things are made of cells. Genes are the part of cells that contain the instructions which tell our bodies how to grow and work, and determine physical characteristics such as hair and eye color. Genes are passed from parent to child.”

“Your tissue sample may be used to create living tissue samples (including cell lines) that can be grown in the laboratory. This allows researchers to have an unlimited supply of your cells in the future without asking for more samples from you. The living tissue samples will be shared with the academic, non-profit research, and for-profit communities. Cancer researchers will use these living tissue samples to study better ways to detect and treat cancer. Companies may use the living tissue samples to develop products that are used to improve the detection and treatment of cancer to benefit patients. The living tissue samples will be sent with the random number corresponding to your original tissue and will not be sent with identifiable health information.”

“Your cells might be used in research involving genetic alteration of the cells. Your cells might be mixed with other human cells, mixed with animal cells, or grown in lab animals like mice. We may also perform a whole genome analysis on the DNA from your clinical samples and living tissue samples. Usually researchers study just a few areas of your genetic code that are linked to a disease or condition. In whole genome studies, all or most of your genes are analyzed and used by researchers to study links to many diseases or conditions.”

“In order to allow researchers to share test results, the National Institutes of Health (NIH) and other central repositories have developed data (information) banks that analyze data and collect the results of whole genome studies. These banks may also analyze and store DNA samples, as well. These central banks will store your genetic information and samples and make them available to the public for additional studies. Because the DNA sequence is available on a database, it is possible that someone using the sequence could identify the donor and possible relatives. The samples and data will be sent with only your code number attached. Your name or other directly identifiable information will not be given to central banks. There are many safeguards in place to protect your information and samples while they are stored in repositories and used for research.”

“Research using your samples, living tissue samples, and their whole genome information is important for the study of virtually all diseases and conditions. Therefore, the sample/data banks will provide study data for researchers working on any disease.”

### HG008 patient history

This dataset is for the first tumor cell line developed from an MGH participant (HG008). The individual, known by the GIAB as HG008, was a 61-year-old with XX sex chromosomes who was diagnosed with pancreatic ductal adenocarcinoma (disease stage ypT2 N1). Resection of PDAC tumor, duodenal tissue, and pancreatic tissue was performed at MGH in December 2020 and sent to the Liss labortatory for tissue banking. After neoadjuvant therapy, the greatest dimension of the tumor was 3.2 cm, located in the pancreatic head near the duodenum. The histologic grade was G2 (moderately differentiated), and the tumor regression grade was 2 (Minimal response: Residual cancer outgrown by fibrosis).

### Establishing the HG008-T tumor cell line

**Figure 1:**
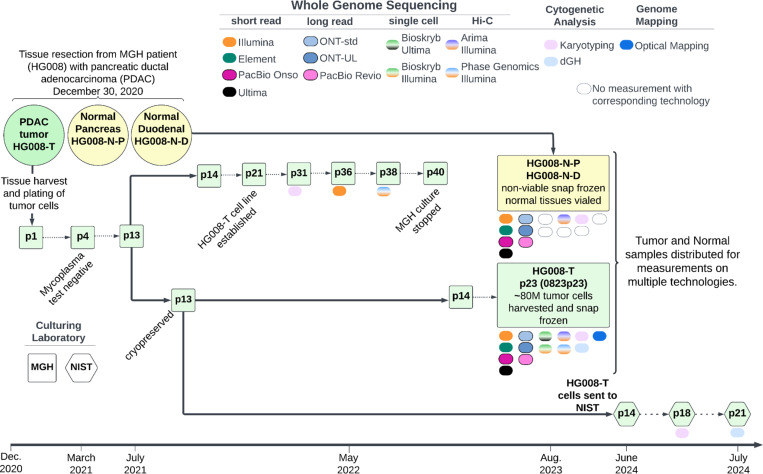
HG008 Passaging and Measurements. The PDAC tumor and normal duodenal and pancreatic tissues were resected from the HG008 individual in 2020. The HG008-T cell line was established by MGH and a bulk growth of tumor cells was produced for most measurements. This bulk growth and harvest is known as batch 0823p23. Tumor and normal samples were sent for measurements on multiple technologies denoted by the colored ovals; empty ovals are where no corresponding measurement was made. To characterize the HG008-T cell line during passaging, preliminary measurements were made and noted below the passage points denoted by “p#”. In 2024, an aliquot of the cell line was transferred to NIST by MGH for additional culturing and measurements.

The Liss laboratory established a PDAC tumor cell line (HG008-T) from the resected PDAC tumor. PDAC tumors are stromal-rich, with cancer cells often only composing 10 % of the tumor. Approximately 50 mg of tumor tissue was enzymatically dissociated to begin culturing from the primary tumor cells for cell line development. While PDAC cell cultures often contain substantial fibroblasts, in this case all cells appeared to be epithelial-like tumor cells even at passage 2 for this cell line. Cells were cultured for the first 20 passages using “Rich” media composed of DMEM/F12 with 20 % Fetal Bovine Serum (FBS), Nicotinamide (0.01 M), cholera toxin (8.4 ng/mL), Epidermal Growth Factor (EGF; 10 ng/mL), Hepatocyte Growth Factor (HGF: 25 ng/mL), human recombinant insulin (10 ug/mL), human recombinant transferrin (5.5 ug/mL), and selenious acid (6.7 ng/mL). Cells were enzymatically removed from the culture surface using 0.25 % trypsin and split by diluting no more than 1:3 for the first 20 passages. Passages 21–24 were diluted 1:3 and grown in the same media as prior passages however without EGF. Morphologically pure cancer cell cultures, free of tumor-associated fibroblasts, were considered cell lines after growing for at least 20 passages. From passage 25 on, the established cell line was then cultured in DMEM/F12 with 10 % FBS. All media contained 1 % antibiotic/antimycotic. A large homogenous batch (batch 0823p23) of tumor cells was cultured and at passage 23, approximately 80 million tumor cells were harvested, pelleted and snap-frozen for distribution and measurement of non-viable cells. Tumor cell line pellets, or isolated DNA from the pellets, and non-viable paired normal tissue, or isolated DNA from the tissue, were distributed for genome-scale measurements as described in Table 2 and Supplementary Table 1. In addition to measurements from the large, homogeneous batch of tumor cells, we also performed karyotyping on p31, Illumina WGS on p36, and Phase Genomics Hi-C on p38, all derived from the same series of passages starting with the p13 stock that batch 0823p23 was grown from. Passage 13 of the tumor cell line was transferred to NIST in summer of 2024 and preliminary measurements of NIST passages p18 and p21 are also presented (Figure 1).

### HG008 paired normal tissues

For the HG008 participant, the laboratory attempted to create an EBV-immortalized lymphoblastoid cell line from frozen PBMCs, but this was unsuccessful. However, several hundred milligrams of normal pancreatic (HG008-N-P) and duodenal (HG008-N-D) tissue was resected, allowing for matched normal sample measurements. These normal sample measurements are primarily to help identify somatic variants in the tumor, but also could be useful for mosaic variant detection within the tissues.

### Tumor and normal samples for measurement

Tumor and normal samples required genomic DNA (gDNA) isolation prior to measurement. In some cases measurement sites chose to perform their own DNA isolation, however, others preferred to receive isolated gDNA. The samples the measurement sites received, either DNA, cells or tissue, are noted in Table 2. For sites receiving gDNA, isolations from non-viable cell pellets of the tumor cell line (batch 0823p23), non-viable normal duodenal tissue and non-viable pancreatic tissue were performed by the Genome Technology laboratory at Northeastern University and distributed to the measurement sites. Methods for measurement sites that performed their own DNA isolations are provided with their measurement methods in the Genome-scale measurements methods section. For sites receiving gDNA, methods for normal tissue and tumor cell line gDNA isolations are provided below and referenced in Genome-scale measurement methods as “previously isolated genomic DNA.”

#### Genomic DNA isolation from normal tissues

Genomic DNA (gDNA) was isolated from two normal tissue types, non-viable pancreatic (HG008-N-P) and duodenal (HG008-N-D), using the Qiagen Puregene Tissue Kit (PN: 158667). Organ tissues, such as the pancreas, contain a significant amount of nucleases therefore care must be taken to minimize gDNA degradation. For this reason, it is expected that gDNA isolation from the pancreas will result in shorter DNA sizing. Approximately 2 mL of Qiagen kit cell lysis buffer with 30 uL of Proteinase K was added to 10–15 mg of tissue. A Dounce Homogenizer was used to disrupt the tissue before leaving it overnight at 55°C. This was followed by addition of RNase A and another incubation at 37°C for 5 minutes followed by cooling on ice. Genomic DNA was precipitated, washed and hydrated in TE buffer (1 mM EDTA, 10 mM Tris·Cl pH 7.5) per the Qiagen Puregene protocol. Two separate isolations were performed for each tissue type and resulting gDNA pooled for each tissue type. For QC, the isolating laboratory measured the yield of gDNA by Qubit and read N50 of gDNA with ONT sequencing. Further, sites performing long-read sequencing independently assessed the gDNA prior to use. Yield, read N50 and sizing are summarized in Table 1. Isolated gDNA from the paired normal tissues was then distributed to various sites for genome-scale measurements as described in Table 2.

#### Genomic DNA isolation from the tumor cell line

Genomic DNA (gDNA) was isolated from non-viable cell pellets of the 0823p23 batch of cells from the HG008-T tumor cell line using the Qiagen Puregene Cell Kit (PN: 158767). Approximately five million cells were input into the Qiagen Puregene protocol. Briefly, cells were lysed, treated with RNase, gDNA precipitated, washed and hydrated in TE buffer (1 mM EDTA, 10 mM Tris·Cl pH 7.5). The gDNA was viscous and was therefore allowed to homogenize for one week at ambient laboratory temperature. Yield of gDNA was measured by Qubit. N50 of gDNA isolated from the tissues was estimated with ONT sequencing and described in Table 1. Isolated genomic DNA from the 0823p23 batch of HG008-T cells was then distributed to various sites for genome-scale measurements as described in Table 2.

### Genome-scale measurements of HG008 tumor and normal samples

Extensive genomic measurements were performed for all HG008 samples; HG008-T, HG008-N-D and HG008-N-P (Figure 1). Here we present twenty GIAB HG008 whole genome-scale datasets. Measurements were performed with thirteen collaborating laboratories representing thirteen distinct measurement technologies (see summary in Table 2 and full description in Supplementary Table 1). The measurement methods presented below include samples measured (HG008-T, HG008-N-P and/or HG008-N-D), DNA isolation, sample preparation, measurement process and validation, as appropriate for the measurement.

#### Bulk short-read whole genome sequencing

##### Illumina NovaSeq 6000

###### PCR-free 2×150 bp Illumina Sequencing (Dataset ID: ILMN-PCR-free-1)

###### DNA Isolation

Genomic DNA (gDNA) was isolated from approximately 10 million non-viable HG008-T cells (passage 36 from 2022 culture) and 80 mg of non-viable HG008-N-P tissue with the Qiagen Gentra Puregene kit using the manufacturer’s instruction. Genomic DNA mass yields for tumor and normal samples were approximately 26 and 9 ug, respectively. Resulting DNA size was quantified using the PicoGreen Assay (Quant-iT^™^ PicoGreen^™^, catalog# P11496) and the quality was assessed using 1 % agarose gel (Invitrogen^™^ E-Gel^™^ Agarose Gels, catalog# G720801).

###### Library Preparation

Library preparation was performed for each sample using an in-house PCR-free protocol, with the Roche Kapa HyperPrep PCR-free Kit (PN: KK8505). Two libraries were prepared for the tumor from 1 ug of gDNA for each library and one library was prepared for the normal duodenal sample from 1 ug of gDNA. For both tumor and normal samples, DNA was sheared into fragments of approximately 450 to 600 bp using a Covaris E220 system. Fragmentation was followed by purification of the fragmented DNA using double-sided bead clean up with different ratios of AMPure XP beads to remove very small and large fragments. DNA end-repair and 3’-adenylation were performed in the same reaction followed by ligation of the barcoded adapters using a set of 96 8-bp barcoded adapters from IDT, Illumina TruSeq RNA unique dual (UD) Indexes (PN: 20040870). Post ligation products were cleaned twice with AMPure XP beads to get rid of leftover adapters/adapter dimers.

###### Whole Genome Sequencing

The resulting tumor and normal PCR-free libraries were sequenced together in one lane using the Illumina NovaSeq 6000 instrument (ICS v1.8.1 and bcl2fastq2 version 2.20.0.422). Paired-end, 2×150 bp sequencing, was performed using the Illumina NovaSeq 6000 S4 Reagent Kit v1.5 – 300 cycles (PN: 20028312) with the NovaSeq XP 4-Lane Kit v1.5 (PN: 20043131).

###### Validation Methods

The Illumina sequencing paired-end FASTQs were assessed with FastQC. The Illumina FASTQs were aligned to the GRCh38 reference genome using BWA-MEM. The GA4GH QC pipeline was run on the resulting BAM files to assess the quality metrics of the alignments (Supplementary Table 2). A summary of QC metrics can be found Table 3.

##### PCR-free 2×150 bp Illumina Sequencing (Dataset ID: ILMN-PCR-free-2)

###### DNA Isolation

Genomic DNA (gDNA) was isolated from approximately five million non-viable HG008-T cells (batch 0823p23) and 40 mg of non-viable HG008-N-D tissue using the Revvity chemagic Prime 8 Instrument. Cells were resuspended and homogenized in the tissue lysis buffer, vortexed, and treated with RNase. The homogenate was then transferred to the chemagic Prime 8 instrument where DNA was extracted using the chemagic Prime DNA Blood 2k Kit H24 kit (PN: CMG-1497). For the normal tissue sample, the tissue was minced with a razor blade, incubated overnight in the lysis buffer with proteinase K. The following day the homogenate was subjected to an RNase treatment and transferred to the chemagic Prime 8 instrument where DNA was extracted using the chemagic Prime DNA blood extraction kit (CMG-1497). Genomic DNA mass yields for tumor and normal samples were approximately 68 and 72 ug, respectively. Resulting DNA size was quantified using the PicoGreen Assay (Quant-iT^™^ PicoGreen^™^, catalog# P11496) and the quality was assessed using 1 % agarose gel (Invitrogen^™^ E-Gel^™^ Agarose Gels, catalog# G720801).

###### Library Preparation

Library preparation was performed for each sample using an in-house PCR-free protocol, with the Roche Kapa HyperPrep PCR-free Kit (PN: KK8505). Two libraries were prepared for the tumor sample from 1 ug of gDNA for each library and one library was prepared for the normal duodenal sample from 1 ug of gDNA. For both tumor and normal samples, DNA was sheared into fragments of approximately 450 to 600 bp using a Covaris E220 system. Fragmentation was followed by purification of the fragmented DNA using double-sided bead clean up with different ratios of AMPure XP beads to remove very small and large fragments. DNA end-repair and 3’-adenylation were performed in the same reaction followed by ligation of the barcoded adapters using a set of 96 8-bp barcoded adapters from IDT, Illumina TruSeq RNA unique dual (UD) Indexes (PN: 20040870). Post ligation products were cleaned twice with AMPure XP beads to get rid of leftover adapters/adapter dimers.

###### Whole Genome Sequencing

The resulting tumor and normal PCR-free libraries were sequenced on an Illumina NovaSeq 6000 (ICS v1.8.1 and bcl2fastq2 version 2.20.0.422). The tumor libraries were pooled with other samples and loaded in a single lane across four flowcells. The normal library was sequenced in a pool of samples and loaded in one lane. Paired-end, 2×150 bp sequencing, was performed using the Illumina NovaSeq 6000 S4 Reagent Kit v1.5 – 300 cycles (PN: 20028312) with the NovaSeq XP 4-Lane Kit v1.5 (PN: 20043131).

###### Validation Methods

The Illumina sequencing paired-end FASTQs were assessed with FastQC. The Illumina paired-end FASTQ data were aligned to the GRCh38 reference genome using BWA-MEM. The GA4GH QC pipeline was run on the resulting BAM files to assess the quality metrics of the alignments (Supplementary Table 2). A summary of QC metrics can be found Table 3.

##### PCR-free 2×150 bp Illumina Sequencing (Dataset ID: ILMN-PCR-free-3)

###### DNA Isolation

Genomic DNA (gDNA) was isolated from approximately two million non-viable HG008-T cells (batch 0823p23) using Qiagen’s QIAamp Blood Mini Kit (PN: 51104), yielding approximately 6.4 ug of gDNA as measured by a Qubit Fluorometer and sizing with peak at approximately 21 kb as measured by an Agilent Fragment Analyzer. HG008-N-D gDNA was previously isolated from non-viable tissue and quality assessed upon receipt, approximately 2.5 ug as measured by a Qubit Fluorometer and sizing with peak at approximately 53 kb as measured by an Agilent Tapestation.

###### Library Preparation

PCR-free library preparation was performed using the Illumina TruSeq DNA PCR-Free High Throughput Library Prep Kit (96 samples) (PN: 20015963), starting with one microgram of gDNA sample. Genomic DNA samples were concentration normalized on a Hamilton robot. Intact gDNA was sheared using the Covaris LE220 sonicator. Fragmentation was followed by an initial cleanup and end-repair, which was then followed by a double-sided bead-based size selection of the repaired DNA fragments. Size selection was followed by A-tailing, in turn followed by ligation using unique dual indexed (UDI) adapters (HG008-T: TTCCTGTT-AAGATACT, HG008-N-D: CGGACAAC-AATCCGGA) to prevent index hopping on patterned flow cells. Two final bead-based clean-ups of the libraries were subsequently performed. The uniquely indexed libraries were then validated using the Fragment Analyzer (Agilent) and pooled based on concentration as determined by Picogreen (ThermoFisher). The final libraries were evaluated for size distribution on the Fragment Analyzer and are quantified by qPCR with adapter specific primers (Kapa Biosystems).

###### Whole Genome Sequencing

The tumor and normal TruSeq libraries were prepared and sequenced approximately four months apart. A single library was prepared for each sample. For each tumor and normal, the TruSeq library was multiplexed and loaded across four lanes of three flow cells to achieve the desired coverage. Paired-end, 2×150 bp sequencing, was performed using the Illumina S4 Reagent Kit v1.5 (300 cycles) (PN: 20028312) on an Illumina NovaSeq 6000 (ICS v1.7.5, RTA v3.4.4).

###### Validation Methods

The Illumina sequencing paired-end FASTQs were aligned to the GRCh38 reference genome using BWA-MEM as part of the NYGC somatic cancer pipeline (https://bitbucket.nygenome.org/projects/WDL/repos/somatic_dna_wdl/browse/README_pipeline.md?at=refs%2Fheads%2Fgiab#deliverables). Prior to alignment, pre-processing of the data was performed using adapter trimming and alignment included short-alignment marking, Novosort duplicate marking, GATK Base quality score calibration (BSQR). The GA4GH QC pipeline was run on the resulting BAM files to assess the quality metrics of the alignments (Supplementary Table 2). A summary of QC metrics can be found in Table 3.

### Element Aviti

#### Short and long insert PCR-free 2×150 bp Aviti Sequencing (Dataset ID: Element-short-2, Element-long-2)

##### Standard (short) Insert Library Preparation

Library preparation was performed using previously isolated genomic DNA (gDNA), as described above in “Tumor and normal samples for measurement”, from non-viable HG008-T cells (batch 0823p23) and non-viable HG008-N-D and HG008-N-P tissue. One short insert library (350 to 400 bp) was prepared from each gDNA source. For each library, 500 ng of gDNA was input into the Element Elevate Mechanical Library Prep Kit (PN: 830–00008, Document # MA-00004 Rev. E) using the Elevate Long UDI Adapter Kit Set A (PN: 830–00010). Mechanical shearing of gDNA was performed using a Covaris ultrasonicator to achieve the desired insert size of approximately 400 bp. For the short insert libraries seven iterations with the following settings were performed: 10 second duration, peak power 50, duty cycle 20, 1000 cycles per burst, average power 10. Shearing was followed by end repair and A-tailing. PCR-free library preparation continued with adapter ligation, generating dual-index libraries. Size selection was performed with double-sided SPRI bead clean-up with SPRI ratios of 0.46X/0.62X. Linear short insert libraries were quantified for loading onto the AVITI system using qPCR.

##### Long Insert Library Preparation

Library preparation was performed using previously isolated genomic DNA (gDNA) from non-viable HG008-T cells (batch 0823p23), as described above in “Tumor and normal samples for measurement”, and non-viable HG008-N-D and HG008-N-P tissue. One long insert library (greater than 1 kb) was prepared from each gDNA source. For each library, one microgram of gDNA was input into the Element Elevate Mechanical Library Prep Kit (PN: 830–00008, Document # MA-00004 Rev. E) using the Elevate Long UDI Adapter Kit Set A (PN: 830–00010). Mechanical shearing of gDNA was performed using a Covaris ultrasonicator to achieve a longer insert size of approximately 800 to 900 bp. For the long insert libraries one iteration with the following settings were performed: 5 second duration, peak power 50, duty cycle 20, 1000 cycles per burst, average power 10. PCR-free library preparation continued with end-repair, A-tailing and adapter ligation, generating dual-index libraries. Size selection was performed with double-sided SPRI bead clean-up with SPRI ratios of 0.3X/0.42X. Linear libraries were quantified for loading onto the AVITI system using qPCR using the 2× Phanta Flash master mix (Vazyme PN: P510) and Evagreen (Biotium PN:31000).

##### Whole Genome Sequencing

Linear libraries were loaded onto individual high output flowcells, 6 flowcells in total (3 for short insert, 3 for long insert). The Element AVITI 2×150 Sequencing Kit Cloudbreak UltraQ sequencing chemistry (PN: 860–00018) was used to generate 150 bp paired-end reads on the AVITI system.

##### Validation Methods

The Element AVITI UltraQ paired-end FASTQs were assessed using FastQC. The FASTQs were aligned to the GRCh38 reference genome using BWA-MEM. The GA4GH QC pipeline was run on the alignment BAM files to assess the quality metrics of the alignments (Supplementary Table 2). A summary of QC metrics can be found Table 3.

### PacBio Onso

#### PCR-free 2×150 bp Onso Sequencing (Dataset ID: PB-Onso-1)

##### Library Preparation

Library preparation was performed using previously isolated genomic DNA (gDNA), as described above in “Tumor and normal samples for measurement”, from non-viable HG008-T cells (batch 0823p23) and non-viable HG008-N-D tissue. PCR-free library preparation was performed using the PacBio Onso fragmentation DNA library prep kit (PN: 102–499–100). Two libraries were prepared, one for each of the tumor (HG008-T) and normal duodenal (HG008-N-D) samples starting with 200 ng of high molecular weight (HMW) gDNA. HG008-T and HG008-N-D HMW genomic DNA (gDNA) were first enzymatically fragmented followed by end-repair, A-tailing and adapter ligation per the fragmentation kit protocol. A small aliquot of libraries were amplified using the PacBio Onso library amp kit (PN: 102–410–800) for library QC purposes (library size measurement by Agilent TapeStation). Quantitative PCR was performed with the PacBio Onso library quant kit (PN: 102–431–800) to quantify Onso-compatible libraries for flow cell loading.

##### Whole Genome Sequencing

Whole genome sequencing of the resulting PCR-free libraries was performed on a PacBio Onso system using sequencing-by-binding (SBB) technology. The tumor library was loaded across four lanes of two flow cells and the normal duodenal library across two lanes of one flow cell. Cluster generation and paired-end 2×150 bp reads were generated using the PacBio Onso 300 cycle sequencing kit (PN: 102–860–300). Onso reverse (R2) reads may contain 1 bp reads. It is advised these reads be filtered out prior to further processing using a suitable trimmer such as cutadapt.8

##### Validation Methods

The PacBio Onso paired-end FASTQs were assessed with FastQC. The FASTQs were aligned to the GRCh38 reference genome using BWA-MEM. The GA4GH QC pipeline was run on the resulting BAM files to assess the quality metrics of the alignments (Supplementary Table 2). A summary of QC metrics can be found Table 3.

### Ultima UG 100

#### PCR-free 300 bp single-end UG 100 Sequencing (Dataset ID: Ultima-bulk-1)

##### Library Preparation

Library preparation was performed using previously isolated genomic DNA (gDNA), as described above in “Tumor and normal samples for measurement”, from non-viable HG008-T cells (batch 0823p23) and non-viable HG008-N-D tissue. Library preparation for each sample, started with 500 ng of isolated genomic DNA. PCR-free library preparation was performed using the NEBNext^®^ Ultra^™^ II FS DNA PCR-free Library Prep Kit (PNs: E7805, E6177) following Ultima Genomics NEB PCR-free WGS Library Preparation Protocol (Publication #: P00014 Rev. D). Ultima Genomics PCR-free adapters, xGen^™^ PCR-free Adapters for Ultima P1, 96 rxn (PN: 10016841) for UG 100 Sequencer compatibility were used.

##### Whole Genome Sequencing

Sequencing was performed on the UG 100 Sequencer (software version : APL 5.1.0.19) using iV27 chemistry. Single-end, 300 bp reads were generated for both samples using the UG_116cycles_Baseline_1.5.3.2 sequencing recipe and flow order [T,G,C,A] on a single wafer. A human genome control sample HG002 (Coriell Nucleic acid ID: NA24385) was spiked into each run in order to ensure quality of the samples being sequenced.

##### Validation Methods

The Ultima single-end FASTQs were aligned to the GRCh38 reference genome using the optimized version of the BWA developed by Ultima Genomics (Supplementary Table 3). The CRAM files were evaluated using the GA4GH Quality control (QC) pipeline to assess the quality metrics of the alignments (Supplementary Table 2). A summary of QC metrics can be found Table 3.

### Hi-C library Preparation

#### Phase Genomics with Illumina sequencing (Dataset ID: HiC-ILMN-1)

##### Library Preparation

CytoTerra library preparation was performed starting with approximately 500,000 viable HG008-T cells (passage 38 from 2022 culture). Proximity ligation preparation was performed with the Phase Genomics Proximo Hi-C v4.5 kit (PN: KT4045) and associated Proximo human protocol (v4.5 0202301, https://4408603.fs1.hubspotusercontent-na1.net/hubfs/4408603/Protocols_latest/Proximo_Human_v4.5_202301.pdf) to generate a dual-indexed Illumina-compatible library. In brief, intact cells are crosslinked, lysed, and chromatin digested with a cocktail of restriction endonucleases. Ends are filled in with biotinylated nucleotides and subjected to proximity ligation. Following ligation, crosslinks are enzymatically reversed and DNA purified. Proximity ligated junctions are purified using streptavidin beads. Bead-bound DNA is used as input for generation of a paired-end Illumina compatible library.

##### Whole Genome Sequencing

The Proximo Hi-C library was run on an Illumina NovaSeq 6000 (ICS v1.7.5, RTA v3.4.4) with 2×150 paired-end reads using the NovaSeq 6000 S4 Reagent Kit v1.5 300 cycles (PN: 20028312). The library was loaded across one lane of a single flowcell.

##### Validation Methods

QC of the HiC data was performed as part of the documented Phase Genomics alignment and QC process (https://phasegenomics.github.io/2019/09/19/hic-alignment-and-qc.html). Reads were aligned to the GRCh38 reference genome using BWA-MEM and resulting alignment QC performed by running the HiC QC python script, hic_qc.py (Supplementary Table 2). QC metrics, used for validation, are further discussed in the Technical Validation section.

#### Arima with Illumina sequencing (Dataset ID: HiC-ILMN-2)

##### HiC Sample Preparation

Arima HiC library preparation used the Arima HiC High Coverage Kit (PN: A410110) following the associated User Guide for Mammalian Cell Lines (PN: Doc A160161 v01, https://arimagenomics.com/wp-content/files/User-Guide-Arima-High-Coverage-for-Mammalian-Cell-Lines.pdf). For each of the HG008-T and HG008-N-D samples, approximately one million were input into the HiC protocol for crosslinking chromatin. The crosslinked chromatin is then digested using a restriction enzyme (RE) cocktail optimized for coverage uniformity across a wide range of genomic sequence compositions. The 5’-overhangs are then filled in, causing the digested ends to be labeled with a biotinylated nucleotide. Next, spatially proximal digested ends of DNA are ligated, capturing the sequence and structure of the genome. The ligated DNA is then purified, producing pure proximally-ligated DNA. The proximally-ligated DNA is then fragmented, and the biotinylated fragments are enriched. HiC ligation resulted in 871 ng HiC ligation product for HG008-T and 1760 ng of HiC ligation product for HG008-N-D.

##### Library Preparation

The HG008-T and HG008-N-D HiC ligation products were prepared for Illumina Sequencing using the Roche KAPA Hyper Prep kit (PN: 7962347001) following the KAPA Hyper Prep kit User Guide (PN: A160139 v00, https://arimagenomics.com/wp-content/files/User-Guide-Library-Preparation-using-KAPA-Hyper-Prep-Kit.pdf). Library preparation begins with DNA fragmentation, DNA size selection, and biotin enrichment. For each sample, 250 ng of sheared and size-selected ligation products were input into a custom Arima-HiC end-repair, dA-tailing and adapter ligation is performed as described in the User Guide.

##### Whole Genome Sequencing

Libraries were shipped to the Baylor College of Medicine Human Genome Sequencing Center (HGSC) for sequencing. The tumor and normal samples were sequenced in a pool of samples in one lane. Paired-end, 2×150 bp sequencing, was performed using the Illumina S4 Reagent Kit v1.5 (300 cycles) (PN: 20028312) on an Illumina NovaSeq 6000 (ICS v1.7.5, RTA v3.4.4). The resulting data is referred to as Arima High Coverage HiC data.

##### HiC Validation

QC was performed as part of the Arima SV pipeline (v1.3, https://github.com/ArimaGenomics/Arima-SV-Pipeline). The pipeline, described here, https://arimagenomics.com/wp-content/files/Bioinformatics-User-Guide-Arima-Structural-Variant-Pipeline.pdf, will run HiCUP^9^ to align the Illumina sequencing reads of the Arima Hi-C library to the GRCh38 reference genome (Supplementary Table 2). The pipeline also produces structural variant calls and visualizations, which, however, are outside the scope of this paper. HiC data QC statistics are then generated for Illumina deeply sequenced data. QC metrics, used for validation, are further discussed in the Technical Validation section.

#### Bulk long-read whole genome sequencing

##### PacBio Revio HiFi

###### PCR-free Revio HiFi Sequencing (Dataset ID: PB-HiFi-1)

###### Library Preparation

Library preparation was performed using previously isolated genomic DNA (gDNA), as described above in “Tumor and normal samples for measurement”, from non-viable HG008-T cells (batch 0823p23) and non-viable HG008-N-P tissue. Sizing of the isolated gDNA was performed prior to library preparation to confirm the gDNA was of sufficient length for long-read sequencing. The average sizing of smear analysis from Pulsed Field Gel Electrophoresis (PFGE) with an Femto Pulse System (Agilent) was approximately 58 kbp for HG008-T and 29 kbp for HG008-N-P.

Library preparation was performed following PacBio procedure and checklist: “Preparing whole genome and metagenome libraries using SMRTbell^®^ prep kit 3.0” (PN: 102–166–600 REV02 MAR2023). This protocol uses the PacBio SMRTbell prep kit 3.0 (PN: 102–182–700), SMRTbell adapter index plate 96A (PN: 102–009–200), and SMRTbell cleanup bead kit (PN 103–306–300). Briefly, 6.4 ug of HG008-T and 6.1 ug of HG008-N-P were sheared separately on a Megaruptor 3 (Diagenode, B06010003) to a target size of 15 to 20 kb. Two library preparations were performed using sheared gDNA inputs of 3.1 ug of HG008-T and 4.1 ug of HG008-N-P. Sheared samples were subjected to a combined DNA repair and A-tailing reaction followed by ligation of sequencing adapters to create SMRTbell libraries. SMRTbell libraries were treated with a nuclease mix to remove unligated library components and then size-selected on a Sage Science PippinHT system using a 0.75 % agarose gel cassette with cassette definition file, “6–10kb High Pass Marker 75E” to remove fragments less than 10 kb.

###### Whole Genome Sequencing

PacBio SMRTbell libraries were prepared for HiFi sequencing (i.e. circular consensus sequencing) by annealing and binding sequencing primers and polymerase, respectively, to the SMRTbell templates using Revio polymerase kit (PN 102–817–600) and following instructions provided in the Sample Setup module of PacBio SMRT Link software (v13.0), a web-based end-to-end sequencing workflow manager. Sequencing-ready SMRTbell libraries were sequenced on a Revio system using the following reagents and consumables: Revio sequencing plate (PN: 102–587–400) and Revio SMRT Cell tray (PN: 102–202–200). 24 hour movies were run. Two SMRT Cells were run for HG008-T and one SMRT Cell for HG008-N-P.

###### Validation Methods

The PacBio Revio unaligned bams were aligned to the GRCh38 reference genome using the PacBio pbmm2 aligner as part of the PacBio HiFi-human-WGS_WDL (germline) pipeline v1.1.0 (https://github.com/PacificBiosciences/HiFi-human-WGS-WDL). Cramino and Nanoplot tools were used to assess the quality of the alignments (Supplementary Table 2). A summary of QC metrics can be found Table 4. While the method used maintains base modification (methylation) information, this was not assessed as part of the data validation process.

###### PCR-free Revio HiFi Sequencing (Dataset ID: PB-HiFi-2)

###### DNA Isolation

Genomic DNA (gDNA) was isolated from approximately five million non-viable HG008-T cells (batch 0823p23) and 40 mg of non-viable HG008-N-D tissue using the Revvity chemagic Prime 8 Instrument. Cells were resuspended and homogenized in the tissue lysis buffer, vortexed, and treated with RNase. The homogenate was then transferred to the chemagic Prime 8 instrument where DNA was extracted using the chemagic Prime DNA Blood 2k Kit H24 kit (PN: CMG-1497). For the normal tissue sample, the tissue was minced with a razor blade, incubated overnight in the lysis buffer with proteinase K. The following day the homogenate was subjected to an RNase treatment and transferred to the chemagic Prime 8 instrument where DNA was extracted using the chemagic Prime DNA blood extraction kit (CMG-1497). Genomic DNA mass yields for tumor and normal samples were approximately 68 and 72 ug, respectively. Resulting DNA size was measured on an Agilent Femto Pulse System with average sizing of approximately 81 kb for gDNA from tumor cell line and 59 kb for gDNA from normal duodenal tissue.

###### Library Preparation

Genomic DNA (7.5ug) was sheared using Covaris g-tubes to achieve an average size of 15 to 20 kb. Sheared DNA was size selected with a Sage Science PippinHT using the 15 to 20 kb high-pass 75E cassette definition (gel cassette HPE7510) to eliminate fragments smaller than 15 kb. One library for each sample type was prepared. The PacBio procedure for library preparation was performed using the PacBio SMRTbell prep kit 3.0 (PN: 102–182–700) and the SMRTbell adapter index plate 96A (PN: 102–009–200) for repair and A-tailing, adapter ligation and nuclease treatment to remove unligated fragments. Final libraries were purified using 1X PacBio SMRTBell Cleanup beads, eluted in 30 μL PacBio elution buffer and quantified using the Qubit dsDNA quantification high-sensitivity assay (Thermo Fisher Scientific). Final library size was determined using the Agilent Femtopulse.

###### Sequencing Methods

PacBio SMRTbell libraries were prepared for HiFi sequencing (i.e. circular consensus sequencing) by annealing and binding sequencing primers and polymerase, respectively, to the SMRTbell templates using Revio polymerase kit (PN: 102–817–600) and following instructions provided in the Sample Setup module of PacBio SMRT Link software (13.0.0.207600). Sequencing-ready SMRTbell libraries were sequenced on a PacBio Revio system (ICS version 13.0.0.212033). 30 hour movies were run. Two SMRT cells each were run for HG008-T and HG008-N-D. QC metrics from SMRTlink software including HiFi reads, mean read length, P1 %, etc were as expected.

###### Validation Methods

The PacBio Revio sequencing unaligned bams (uBAM) raw data were aligned to the GRCh38 reference genome using the PacBio pbmm2 aligner as part of the PacBio Somatic variant calling Pipeline v0.7 (https://github.com/PacificBiosciences/HiFi-Somatic-WDL). Cramino and Nanoplot tools were used to assess the quality of the alignments (Supplementary Table 2). A summary of QC metrics can be found Table 4. While the method used maintains base modification (methylation) information, this was not assessed as part of the data validation process.

### Oxford Nanopore Technologies PromethION

#### PCR-free Standard Length PromethION Sequencing (Dataset ID: ONT-std-1)

##### Library Preparation

Library preparation was performed using previously isolated genomic DNA (gDNA), as described above in “Tumor and normal samples for measurement”, from non-viable HG008-N-P and HG008-N-D tissues. Library preparation was performed with Oxford Nanopore Technologies Ligation Sequencing Kit V14 (PN: SQK-LSK114) using one microgram of gDNA from each tissue.

##### Whole Genome Sequencing

Nanopore based sequencing was performed on Oxford Nanopore Technologies (ONT) PromethION utilizing ONTs PromethION R10.4.1 Flow Cells (PN: FLO-PRO114M). To maximize throughput multiple flow cells were used. Sequencing was performed using four flow cells for the duodenal sample and three flow cells for the pancreatic sample.

##### Basecalling

The Dorado v0.5.3 basecaller (https://github.com/nanoporetech/dorado) and DNA model ‘dna_r10.4.1_e8.2_400bps_sup@v4.3.0_5mC_5hmC@v1’ were used for basecalling, including modified bases. Basecalling successfully completed with no errors. Output from the basecaller is a bam file of unaligned reads including modified bases.

##### Validation Methods

The Oxford Nanopore Technologies Dorado basecaller generates a ‘summary.txt’ file, which was analyzed using PycoQC to assess sequencing quality metrics.The Oxford Nanopore Technologies sequencing unaligned bams were processed to extract the FASTQ sequences by using samtools then aligned to the GRCh38 reference genome using the Minimap2. Cramino and Nanoplot tools were used to assess the quality of the alignments (Supplementary Table 2). A summary of QC metrics can be found Table 4. While the method used maintains base modification information, this was not assessed as part of the data validation process.

#### PCR-free Standard Length PromethION Sequencing (Dataset ID: ONT-std-2)

##### DNA Isolation

DNA was extracted from approximately five million non-viable HG008-T cells (batch 0823p23) utilizing the NEB Monarch^®^ HMW DNA Extraction Kit for Tissue (PN: T3060). Total DNA extracted was approximately 80 μg. Only 5 μg of DNA was sheared using Diagenode’s Megaruptor3 (PN: B06010003) with the DNAFluid+ Kit (PN: E07020001) followed by DNA size selection using PacBio SRE kit (PN: 102–208–300). The size of sheared DNA fragments were analysed on Agilent Femto Pulse System using genomic DNA 165 kb kit (PN: FP–1002–0275). Fragment size distribution of post-sheared DNA had peak at approximately 45 kb length.

##### Library Preparation

The UCSC protocol had been optimized to generate N50s of approximately 30 kb with very high throughput for ligation based sequencing. Library preparation was performed with Oxford Nanopore Technologies Ligation Sequencing Kit XL V14 (PN: SQK-LSK114-XL). Enough library was created for 4 library loads onto flow cells.

##### Whole Genome Sequencing

Nanopore based sequencing was performed on Oxford Nanopore Technologies (ONT) PromethION 48/PromethION A Series Data Acquisition Unit (PN: PRO-SEQ048/PRO-PRCAMP) utilizing ONT PromethION R10.4.1 Flow Cells (PN: FLO-PRO114M). Sequencing was performed on the same day as ligation library preparation. Sequencing was performed utilizing one PromethION R10.4.1 Flow Cell. The flow cell was washed with ONTs Flow Cell Wash Kit XL (PN: EXP-WSH004-XL) each day and re-primed and loaded with fresh library for 4 days of sequencing to maximize throughput.

##### Basecalling

The Dorado v0.3.4 basecaller (https://github.com/nanoporetech/dorado) and model ‘dna_r10.4.1_e8.2_400bps_sup@v4.2.0/dna_r10.4.1_e8.2_400bps_sup@v4.2. 0_5mCG_5hmCG@v2’ were used for basecalling, including modified bases. Basecalling successfully completed with no errors. Output from the basecaller is a bam file of unaligned reads including modified bases.

##### Validation Methods

The Oxford Nanopore Technologies Dorado basecaller generates a ‘summary.txt’ file, which was analyzed using PycoQC to assess sequencing quality metrics. The Oxford Nanopore Technologies sequencing unaligned bams were processed to extract the FASTQ sequences then aligned to the GRCh38 reference genome using the Minimap 2. Cramino and Nanoplot tools were used to assess the quality of the alignments (Supplementary Table 2). A summary of QC metrics can be found Table 4. While the method used maintains base modification (methylation) information, this was not assessed as part of the data validation process.

#### PCR-free Ultra Long PromethION Sequencing (Dataset ID: ONT-UL-1)

##### DNA Isolation

Ultra-high molecular weight DNA was extracted from approximately five million non-viable HG008-T cells (batch 0823p23) utilizing New England Biolabs Monarch^®^ HMW DNA Extraction Kit for Tissues (NEB #T3060). Wide bore pipette tips were used throughout to preserve ultralong DNA. No QC of resulting gDNA was performed as viscosity of isolated DNA solution is challenging to work with. Rather ultra long DNA is assessed as part of the ONT sequencing and previous experience with a five million cell as input.

##### Library Preparation

Library preparation was performed utilizing Oxford Nanopore Technologies Ultra-Long DNA Sequencing Kit Version 14 (SQK-ULK114). The library was eluted in approximately 810 μL volume in order to sequence across additional flow cells to maximize data collection.

##### Whole Genome Sequencing

Nanopore based sequencing was performed on an Oxford Nanopore Technologies (ONT) 48/PromethION A-Series Data Acquisition Unit (PN: PRO-SEQ048/PRO-PRCAMP) utilizing ONTs PromethION R10.4.1 Flow Cells (PN: FLO-PRO114M). Sequencing was performed the day following library prep utilizing three PromethION R10.4.1 Flow Cells. The flow cells were washed with ONTs Flow Cell Wash Kit XL (EXP-WSH004-XL) each day and reloaded with the library for 4 days of sequencing to maximize throughput.

##### Basecalling

The Dorado v0.4.3 basecaller (https://github.com/nanoporetech/dorado) and model ‘dna_r10.4.1_e8.2_400bps_sup@v4.2.0/dna_r10.4.1_e8.2_400bps_sup@v4.2. 0_5mCG_5hmCG@v2’ were used for basecalling, including modified bases. Basecalling successfully completed with no errors. Output from the basecaller is a bam file of unaligned reads including modified bases.

##### Validation Methods

The Oxford Nanopore Technologies Dorado basecaller generates a ‘summary.txt’ file, which was analyzed using PycoQC to assess sequencing quality metrics.The Oxford Nanopore Technologies sequencing unaligned bams (uBAM) were processed to extract the FASTQ sequences by using samtools then aligned to the GRCh38 reference genome using the Minimap2. Cramino and Nanoplot tools were used to assess the quality of the alignments (Supplementary Table 2). A summary of QC metrics can be found Table 4. While the method used maintains base modification (methylation) information, this was not assessed as part of the data validation process.

#### Single cell whole genome sequencing

##### BioSkryb ResolveDNA with Illumina Low Pass Sequencing (Dataset ID: sc-ILMN-1)

###### Whole Genome Amplification (WGA)

Approximately two million non-viable HG008-T cells (batch 0823p23) were sorted with a Sony SH800 using a 130 micron chip. Singlet (FSC-A / FSC-H, BSC-A / BSC-W) and live-cell (PI negative, top 70 % Calcein-AM positive) gating was employed for single cell sorting into a 384-well plate with 3 μL of BioSkryb Cell Buffer (PN: 100199). Individual cells were processed within wells using the ResolveDNA WGA Kit (PN: 100136) following steps outlined in the ResolveDNA Whole Genome Amplification Kit v2.0 User Guide. Primary Template Directed Amplification (PTA) to amplify single cell whole genomes and transform into Illumina-sequenceable libraries. Approximately 70 % of cells met specifications for BioSkryb WGA metrics. Dual-indexed Illumina-compatible libraries were generated for the top performing 120 cells.

###### Illumina Low-Pass Sequencing

Illumina low pass sequencing performed to QC the single cell libraries. libraries were pooled and sequenced on a single flowcell targeting 2M reads as an initial QC using an Illumina NextSeq 1000 instrument with 2×50 paired-end reads with P2 reagents v3 (PN: 200446811).

###### Single Cell Library Validation

Single-cell libraries were assessed by running BioSkryb’s BJ-DNA-QC pipeline (https://docs.basejumper.bioskryb.com/pipelines/secondary/bj-dna-qc/1.8.2/docs/#background) through the BioSkryb analytics platform, BaseJumper. The BJ-DNA-QC pipeline uses the Illumina low-pass sequencing data and generates several QC metrics that help assess whether the single-cell libraries are ready for high-depth sequencing. Briefly, reads were aligned to the GRCh38 reference and QC-metrics obtained from the derived alignment, and are used to censor single-cells. QC metrics, used for validation, are further discussed in the Technical Validation section (Supplementary Table 2).

##### BioSkryb ResolveDNA with Ultima High Throughput Sequencing (Dataset ID: sc-Ultima-1)

###### Library Conversion

BioSkryb Genomics previously prepared 120 ResolveDNA single cell libraries for Illumina low-coverage sequencing; described above for sc-ILMN-1 dataset. For high-coverage Ultima sequencing these libraries were then converted to a format compatible with the Ultima Genomics sequencing platform (UG 100), following the Ultima Genomics Library Conversion protocol in UG Library Amplification Kit v3 User Guide (Publication #: P00025 Rev. B). Library Amplification followed option 2 for starting with non-UG adapters. The UG Library Amplification Kit v3 (supplied by Ultima Genomics) and the IDT kit xGen^™^ Indexing Primers for Ultima (PNs: 10016992 and 10016993) were used for conversion and enabled multiplexing of the single cell libraries. The library amplification step was modified to replace the UG Index Primer and UG Universal primer with 5.0 μL of primer mix from the appropriate well of the IDT index primer plate. Ten nanograms of input library was used for each reaction and seven cycles of PCR were performed per the amplification protocol. Final converted libraries were QC’d for size and yield using an Agilent Tapestation and Qubit, respectively. Two plates of 120 individual Ultima single-cell libraries were sent to Ultima Genomics for sequencing.

###### Whole Genome Sequencing

Sequencing of the converted (Illumina to Ultima) libraries was performed on the UG100 (software version : APL 5.1.0.19) using V27 Chemistry. Single end, 300 bp reads were generated using the UG_116cycles_Baseline_1.5.3.2 sequencing recipe and flow order [T,G,C,A] on a single wafer. A human genome control sample HG002 (Coriell Nucleic acid ID: NA24385) was spiked into each run in order to ensure quality of the samples being sequenced.

###### Validation

To generate sequencing measures and QC statistics, a custom Ultima tool is run that implements select picard metrics https://github.com/broadinstitute/picard. This tool collects the metrics on board of the UG100 sequencing platform in parallel with producing sorted, duplicated-marked GRCh38 aligned CRAM files. Alignments are produced using an Ultima-optimized version of BWA-MEM. QC metrics, used for validation, are further discussed in the Technical Validation section (Supplementary Table 2).

#### Whole genome optical mapping

##### Optical Mapping with Bionano Saphyr (Dataset ID: Bionano-1)

###### DNA Isolation

Approximately two million non-viable HG008-T cells (batch 0823p23), were washed with Bionano Stabilizing Buffer (PN: 20394 and 20397) by MGH at the time of pelleting and freezing. Upon receipt of the cell pellet, ultra-high molecular weight genomic DNA (gDNA) was isolated using the Bionano SP-G2 Blood & Cell Culture DNA Isolation Kit (PN: 80060) with a yield of approximately 6.5 ug of gDNA (100 ng/μL in 65 μL elution) with an N50 greater than 150 kbp.

###### Sample Preparation

Sequence-specific labeling of the gDNA was performed using the Bionano Direct Label and Stain-G2 (DLS-G2) Kit (PN: 80046).

###### Optical Genome Mapping

Labeled DNA was loaded onto a Bionano Saphyr Chip G3.3 and measured with the Saphyr System (ICS v5.3.23013.1). Molecules and labels imaged on the Saphyr instrument were digitized and saved in a BNX format (raw data) (https://bionanogenomics.com/wp-content/uploads/2018/04/30038-BNX-File-Format-Specification-Sheet.pdf) which served as input into downstream analyses. The Rare Variant Pipeline (RVP) (https://bionano.com/wp-content/uploads/2024/04/CG-30375-Analysis-Quick-Start-Annotated-Rare-Variant-Structural-Variant-Calling.pdf) detected SVs by the “split-read” analysis, where initial molecule alignment and molecule extension refinement allows identification and detection of somatic variants, and identifies variants that are 5 kbp or longer in size. The Access software (v1.8) was used to generate high confidence callsets for SVs and unique SVs not present in the Bionano internal control database. High confidence CNV calls were generated after recentering based on the hypodiploid tumor genome. Regions where unique SV and CNV calls overlap with Bionano custom pan cancer gene list are also reported.

###### Validation

This sample was processed based on manufacturer protocol.^10^, QC metrics, used for validation, are further discussed in the Technical Validation section (Supplementary Table 2).

#### Cytogenetic analysis

##### G-Banded Karyotyping (MGH p31 karyotyping-1 and NIST p18 karyotyping-2)

**Figure 2:**
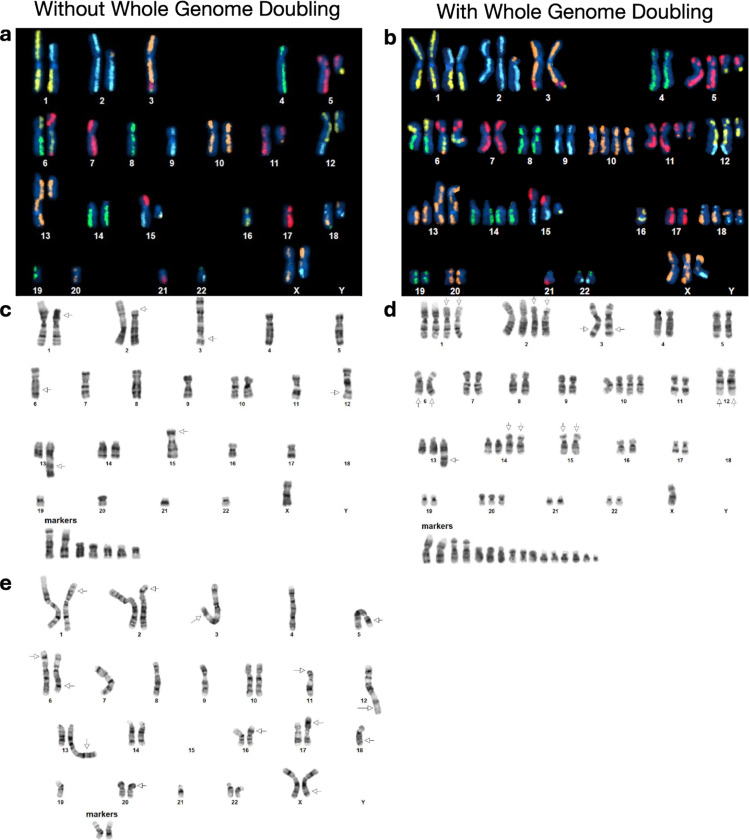
Directional Genomic Hybridization (dGH) and G-banded karyograms for three passages of the HG008-T tumor cell line. Karyograms show examples of cells with and without whole genome doubling. **(a-b)** dGH karyograms from NIST passage 21 (2024) have each chromosome colored by one of five dyes, and information from G-banded karyograms and copy number alterations were used to make preliminary assignments of chromosomes. **(c-d)** G-banded karyotypes from MGH passage 31 (2022) with and without whole genome doubling (**e**) G-banded karyotype from NIST passage 18 (2024) without whole genome doubling, representative of 17 of 20 spreads that had 34 to 36 chromosomes. While not shown, three out of twenty spreads showed whole genome doubling, with counts ranging from 68 to 70 chromosomes.

Karyotyping was performed by KaryoLogic Inc (Durham, NC) for two passages of HG008-T as shown in Figure 2. Approximately one million viable HG008-T cells were plated in T25 cell culture flasks, two from MGH and one from NIST, and sent for karyotyping. Cytogenetic analysis was performed on twenty-five G-banded metaphase spreads of the HG008-T cell line from MGH passage 31. A second analysis was performed in 2024 using viable HG008-T cells from NIST passage 18. Cytogenetic analysis was performed on twenty G-banded metaphase spreads for the HG008-T NIST passage 18 cells (2024).

##### Kromatid Directional Genomic Hybridization (dGH) (Dataset ID: dGH-1)

###### Sample Preparation

Sample preparation for dGH analysis was performed at NIST from passage 21 of HG008-T. One million cells were plated in a T25 flask and were cultured overnight. The cell culture medium was replaced with dGH cell prep kit (KromaTiD, Cat# dGH-0001) contained culture medium, and additional dose of dGH cell prep kit was added 4 hr after medium replacement. Cells were arrested in the first mitosis with a 4-hour Colcemid (KromaTiD, Cat# COL-001) block, harvested, fixed in freshly made fixative [3:1 methanol:acetic acid (Fisher Scientific, Waltham, MA, USA)], and shipped to KromaTiD. Metaphase spread preparation along with subsequent UV and exonucleolytic treatments to selectively remove the analog-incorporated daughter strand were performed at KromaTiD according to published dGH protocols.^11^

###### Directional Genomic Hybridization

For whole genome analysis, KromaTiD’s dGH SCREEN assay was hybridized to the prepared metaphase spreads, then counterstained with DAPI (Vectashield, Vector Laboratories, Newark, CA, USA) and imaged on an Applied Spectral Imaging Harmony system (Applied Spectral Imaging, Carlsbad, CA, performed USA) using a 100X objective. The dGH SCREEN assay consists of single-stranded, unidirectional, tiled oligos designed to hybridize directly to the unique sequences in each of the 24 human chromosomes. Each chromosome specific complement of oligos is end-labeled with one of five unique spectrally differentiable fluorophores, and each of these “high-density chromosome paints” are combined such that chromosomes painted in the same color can be differentiated by size, shape, and centromere position. Prior to analysis, images of dGH SCREEN painted metaphase spreads are qualified, processed and sorted into karyograms for rapid, consistent reading of the assay.

A combination of expected dGH SCREEN signal patterns in the (diploid) human reference genome,^12^ the HG008-T standard G-banding karyotypes, and the copy number variant was used to construct preliminary dGH SCREEN karyograms in 10 cells, including two (2) genome-doubled cells. The prototype karyograms will be used to generate per-chromosome attribution of inter- and intra-chromosomal structural variation events including inversions, translocations, aneuploidy (gain and loss), insertions, centromere abnormalities and complex events across the sample in additional cells from passage 21 (Figure 2).

#### Somatic variant annotation

An initial annotation of a publicly available HG008-T somatic variants called by DRAGEN v.4.2.4 (https://ftp.ncbi.nlm.nih.gov/ReferenceSamples/giab/data_somatic/HG008/Liss_lab/superseded-2022-data/analysis/DRAGEN-v4.2.4_ILMN-WGS_20230914/dragen_4.2.4_HG008_tumor.hard-filtered.vcf.gz.) was performed using ANNOVAR (v2020–06–08, https://annovar.openbioinformatics.org/en/latest/). DRAGEN calls were made from the Illumina-PCR-free-1 dataset. ANNOVAR was run using databases and annotation datasets: refGene, ClinVar (v20221231 and v20220320), Catalogue Of Somatic Mutations In Cancer (COSMIC) (v92, https://cancer.sanger.ac.uk/cosmic), gnomAD (v2.1), 1000 Genomes Project (2015 Aug), and the Exome Sequencing Project (ESP), as well as curation of genes commonly mutated in PDAC tumors.

#### HG008 ancestry analysis

We examined global genetic ancestry of the HG008 tumor and normal pancreatic samples, HG008-T and HG008-N-P (tissue) respectively, utilizing SNVs called by xAtlas from the Illumina-PCR-free-1 dataset. Ancestry was assessed by germline genetic similarity of these “target samples” to reference samples of known genographic origin from the 1000 Genomes Project (Phase 3).^13^ For computational efficiency, we limited our analysis to chromosome 21. We used PLINK^14^ (http://pngu.mgh.harvard.edu/purcell/plink/) to restrict our analysis to SNPs with minor allele frequency greater than or equal to 5 % in the reference panel. We then extracted these overlapping loci from the target sample Variant Call File (VCF) and then merged the datasets into a single VCF with bcftools.^15^ During the merging step, we assumed that entries absent from the target sample VCF at these sites were homozygous for the reference allele. We then performed principal component analysis (PCA) using PLINK and visualized genetic similarity on the first three principal component axes.

#### Haplotype-specific copy number analysis

Haplotype-specific copy number analysis was performed using Wakhan (https://github.com/KolmogorovLab/Wakhan; manuscript in preparation). Wakhan computes haplotype-specific coverage of the genome using phased heterozygous germline variants. Then it identifies boundaries of the copy number alteration (CNA) events by analysing SV breakpoints. Prior to running Wakhan, we phased the germline variants with PacBio long-read (Dataset ID: PB-HiFi-1) and Hi-C (HiC-ILMN-2) data using HiPhase^16^ and HapCUT2.^17^

## Data Records

The data presented were collected as part of Genome in A Bottle Consortium efforts. This data and alignments are made public on the GIAB FTP site hosted by the NIH-NCBI (https://ftp.ncbi.nlm.nih.gov/ReferenceSamples/giab/data_somatic/HG008/), as well as Amazon cloud (s3://giab/data_somatic/HG008/). Future data and analyses collected for the GIAB HG008 sample will be made available on the GIAB FTP as a public resource. Sequencing data can also be browsed through 42basepairs (https://42basepairs.com/browse/web/giab/data_somatic/HG008/), which allows for high level exploration of the data.

In addition, we are working to make all sequencing data presented publicly accessible in the SRA database under NCBI BioProject PRJNA200694. Full dataset descriptions are included in Supplementary Table 1 and include measurement technology, institution, sample source, sample measured, e.g. cell line batch/passage and associated file types. A manifest of all files for the datasets with filenames, md5 checksums, and data citations are provided in Supplementary File 1.

## Technical Validation

### The tumor cell line has many characteristics of a typical tumor

To get a general overview of the aneuploidy and rearrangements in the tumor cell line, standard karyotyping was performed for MGH passage 31 (2022) and NIST passage 18 (2024) as well as karyotyping using dGH for NIST passage 21 (2024) of the tumor cell line; Dataset IDs karyotyping-1, karyotyping-2 and dGH-1, respectively. The karyotyping results indicate a subpopulation of the cell line has undergone whole genome doubling (Figure 2). Whole genome doubling generally is difficult to detect with bulk WGS, but is a common result of *TP53* loss, as discussed below.^18^

**Figure 3:**
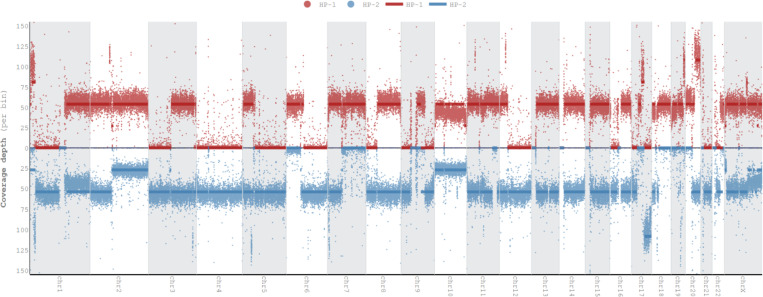
Haplotype Specific Coverage Plot. Plot shows coverage of haplotype 1 (HP-1, red) and haplotype 2 (HP-2, blue) for all chromosomes using HG008-T HiFi reads visualized using Wakhan (https://github.com/KolmogorovLab/Wakhan).

To give an overview of copy number alterations in HG008-T, Figure 3 depicts the coverage by reads from each haplotype. The cell line appears to have many large deletions that cause loss of heterozygosity, with fewer duplications. However, whole genome doubling in some cells results in four copies of regions without deletions and two copies of regions with deletions.

We performed an initial annotation of somatic variants (https://ftp-trace.ncbi.nlm.nih.gov/ReferenceSamples/giab/data_somatic/HG008/Liss_lab/superseded-2022-data/analysis/DRAGEN-v4.2.4_ILMN-WGS_20230914/annotations/dragen_4.2.4_HG008_tumor.hard-filtered.hg38_multianno_clinvar.vcf.gz) and focused on somatic variants in genes commonly mutated in PDAC tumors. The cell line contains the *KRAS* [c.35G>T;p.G12V] (COSMIC ID: COSV55497419, and Pathogenic in ClinVar https://www.ncbi.nlm.nih.gov/clinvar/variation/12583/) gain of function mutation common in PDAC^19^, as well as a likely duplication of the copy of the gene containing this mutation. In addition, there is a 20 kbp deletion of part of the *p16*/*CDKNA2* gene, and the other haplotype was lost due to a very large deletion. Other small somatic mutations that may have a functional impact on genes associated with cancer include the mutation K132T [c.356C>G;p.Ala119Gly] in the remaining copy of the tumor suppressor gene *TP53* (COSV52759171, and Likely Pathogenic in ClinVar https://www.ncbi.nlm.nih.gov/clinvar/RCV001220864/),^20^ and a frameshift mutation in the tumor suppressor gene *SMAD4* associated with resistance to therapy.^21^ Therefore, HG008-T has somatic mutations in the four most commonly mutated genes in TCGA’s analysis of 150 PDAC tumors.^22^ Additional stopgain somatic mutations in HG008-T and COSMIC include a SNV mutation in *GLP2R* [c.1030C>T, p.R344*] (COSV52327429), and a SNV mutation in *INSYN1* [c.457C>T, p.R153*] (COSV65838057). Note that these are examples of potentially important somatic mutations in HG008-T, all of which appear to be in all tumor cells and not in normal cells, but are not a comprehensive list. Also, it is possible that some of these may not have been in all cells in the primary tumor and may be new mutations that occurred during cell line development. In addition, we compared the large-scale copy number alterations observed in HG008-T to recently published signatures of somatic copy alterations in TCGA samples,^23^ and the locations and prevalence of large deletions are similar to the chromosomal-scale loss of heterozygosity (cLOH) signature. The large-scale deletions followed by whole genome doubling in some cells is also consistent with the known progression of *TP53*-deficient tumors.^18^

### HG008 genetic ancestry analysis

**Figure 4:**
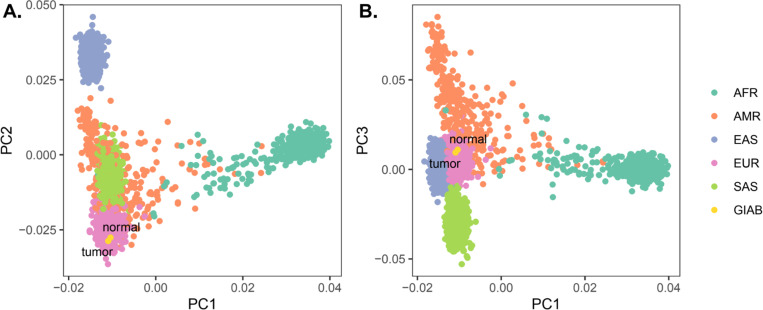
Ancestry Principal component analysis (PCA) Plot for HG008 using continental superpopulations of the 1000 Genomes reference samples. The PCA uses PLINK to visualize clusters of genetic similarity, and we show the first three principal component axes, which distinguish the superpopulations. Yellow points indicate both the normal and tumor cells’ variants from the HG008 individual make them most similar to individuals with European ancestry based on the first three principal components.

To assess genetic ancestry of the HG008 individual, we performed principal component analysis (PCA) using PLINK and visualized genetic similarity on the first three principal component axes (Figure 4). The tumor and normal sample pair exhibited high genetic similarity on these axes, supporting their common germline origin, while also exhibiting the greatest genetic similarity to 1000 Genomes reference samples^13^ from the European superpopulation.

### PCR-free whole genome sequencing validation

To validate the quality of the sequencing data, we generated common sequencing QC metrics with public tools before and after mapping reads to the GRCh38-GIABv3 reference genome. We leveraged the GA4GH WGS quality control standard reference implementation (https://www.ga4gh.org/product/wgs-quality-control-standards/) for bulk WGS short-read data (Table 3). When appropriate comparable QC tools were used for bulk WGS long-read data (Table 4). More information on specific tools and metrics generated for validation of short-read and long-read data can be found in the Supplementary Table 2 (Validation Tools) and Supplementary Table 3 (Metrics Descriptions). Overall, the QC metrics and data characteristics were consistent with expectations and the data were of sufficient quality for downstream analysis and use in material characterization. Additionally, the datasets have already been used for multiple downstream analyses, e.g. variant calling and genome assembly, further validating the data quality.

Because the tumor cell line is aneuploid, we also estimate the mean coverage of apparently diploid and haploid regions, as well as number of reads per tumor chromosomal copy (NRPCC).^23^ For these estimations, we use the mean coverage of chromosome 4 as the haploid coverage because it appears to be haploid in most or all cells, as was observed in the single cell data (Dataset ID sc-ILMN-1). We calculate the diploid coverage as two times the haploid coverage and NRPCC as half the haploid coverage. The diploid and haploid coverages are estimated for cells without whole genome doubling, whereas NRPCC is the coverage of each copy of the chromosome for cells with whole genome doubling.

A summary of metrics generated by GA4GH Quality control pipeline for short-read sequencing technologies Ultima, Onso, Illumina, and Element are presented in Table 3. The short-read metrics (Table 3) show the insert sizes mean and standard deviations were consistent with expected values for the library preparation methods used.

For long-reads, Table 4 has similar metrics for coverage but has some different metrics due to differences in QC tools used for long versus short-reads. For long-read technologies including ONT-std, ONT-UL, and Pacbio-Revio, QC tools such as Cramino and NanoPlot provide long-read specific metrics for validation (Supplementary Table 2 (Validation Tools) and Supplementary Table 3 (Metrics Descriptions)). PycoQC metrics for the ONT datasets were generally consistent with expectations, with one exception. The first flowcell run for the ONT-std-1 dataset showed slightly lower read quality, Q18 compared to Q21 for subsequent flowcells measuring the pancreas library, as reported by pycoQC. This is likely a result of flowcell variability and the flowcell being overloaded for the initial run, approximately 1.5X higher. For the two subsequent flowcells using the pancreas library, the DNA mass input was adjusted down.

**Figure 5:**
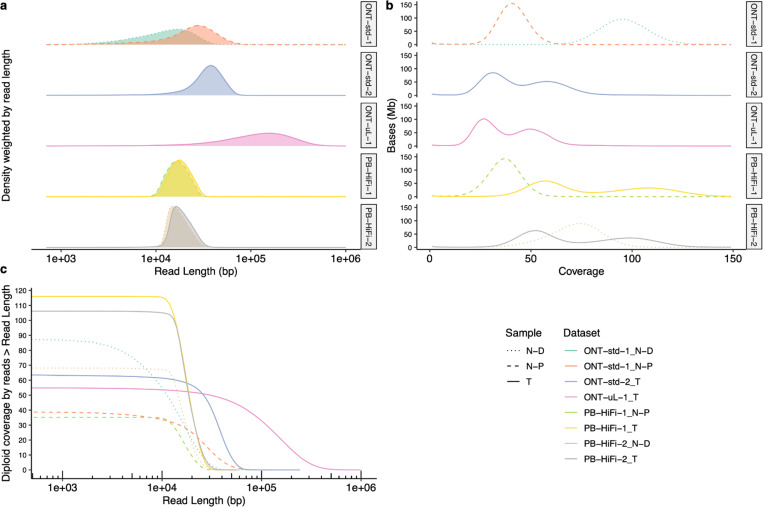
Long-read Coverage Plots. Read length and coverage distributions for long-read datasets **(a)** Mapped read length distribution weighted by aligned read length. **(b)** Coverage distribution showing number of genome positions (Mb) at each integer coverage. **(c)** Inverse cumulative distribution showing coverage of apparently diploid regions by aligned reads longer than the length on the x axis (this assumes no whole genome doubling)

Figure 5a shows the read length distributions weighted by read length, similar to the N50 metric, and shows that ONT reads (particularly ultra long ONT, ONT-UL) are substantially longer than HiFi for the tumor cell line. However, because extracting very long DNA from normal tissues is more challenging than cell lines, most ONT reads from normal pancreatic and duodenal tissue are shorter than from the tumor cell line. Figure 5c depicts this information in a different manner to make it easier to see the coverage by reads longer than a certain length (e.g., there is 33× coverage by ONT-UL reads longer than 100 kb). Figure 5b shows the coverage has a single peak for normal cells but has two peaks for tumor cells due to apparently haploid and diploid regions.

### Additional genome-scale measurement validation

For other datasets, QC metrics specific to each data type were generated and used to validate the datasets. The methods used for each validation are described in the associated Methods section above and in Supplementary Table 2.

#### Optical mapping validation

##### Bionano

All quality metrics for the Bionano-1 dataset passed manufacturer’s recommendations. The HG008-T sample had N50 of 322.82 kbp, labelling density (label/100 kbp) of 16.26, map rate of 92.2 % and effective coverage of 434.72X.

#### BioSkryb single cell library validation

##### Illumina library validation

Single-cell libraries, from dataset sc-ILMN-1, were validated using the metrics reported by BioSkryb analytics BaseJumper platform using the BJ-DNA-QC pipeline (https://docs.basejumper.bioskryb.com/pipelines/secondary/bj-dna-qc/1.8.2/docs/#background). The BioSkryb BJ-DNA-QC pipeline metrics include genome coverage, percentage of mitochondrial contaminating reads, percentage of chimeric reads and assessing diversity of reads. A description of the BaseJumper reported metrics and their criteria for acceptance can be found in Supplementary Table 3. Pre-seq counts are the estimation of the genomic coverage of a given library if it were to be run at the WGS level. In biological samples, pre-seq counts less than 3,000,000,000 can also represent biological genomic alterations (such as chromosomal losses). Samples with less than 3 billion pre-seq that meet other criteria are manually reviewed for copy number alteration to determine if biological features may be impacting this value. Because HG008-T contains substantial copy number alterations, particularly chromosomal losses, we are publishing data from cells that have pre-seq counts less than 3 billion.

Based on the metrics described above, a total of 98/120 libraries met the specifications by BioSkryb Genomics quality control. However, sequencing data for all 120 libraries is provided because the 22 libraries that did not pass BioSkryb QC might still be high quality libraries for cells with significant copy number alterations. Metrics for all 120 single-cell libraries are reported in Supplementary File 2, BioSkryb BJ-DNA-QC.

### Ultima library validation (Dataset ID: sc-Ultima-1)

Ultima sequencing of the BioSkryb single cell libraries, presented in dataset sc-Ultima-1, was validated using the QC metrics reported from the UG100 sequencing run in Supplementary File 2, BioSkryb Ultima QC. Data is considered acceptable if the percentage of aligned reads is greater than 99 %, percentage of bases with a base quality of 20 is greater than 90 %, and the indel rate is less than 0.7 %. Library metrics were evaluated and 119/120 single cell libraries passed acceptance criteria. One library failed to meet acceptance criteria and was removed from the dataset.

#### HiC library validation

##### Phase Genomics

The HG008-T CytoTerra library, presented in dataset HiC-ILMN-1, was validated using the Phase Genomics hiC_QC.py script (https://github.com/phasegenomics/hic_qc). The library met all Phase Genomics CytoTerra QC acceptance criteria as described in Supplementary Table 3. High-quality read pairs are those having MAPQ greater than zero, are not PCR duplicates and map to different contigs or are greater than10 kb apart. The percentage of high-quality read pairs where both reads map to the same strand in the reference genome is 47.54 %, well exceeding the minimum acceptable criteria of 15 %. The fraction of high-quality read pairs greater than 10 kb apart is 75.27 %, exceeding minimum acceptable criteria of 20 %. The fraction of duplicate reads is 21.28%, below maximum acceptable criteria of 40 %. Finally, the frequency which pairs of reads map to different contigs in an assembly (intercontig read pairs) is 22.25 %, below the maximum acceptance criteria of 30 %.

##### Arima Genomics

The HG008-T and HG008-N-D HiC libraries and resulting sequencing, presented in the HiC-ILMN-2 dataset, were found to meet criteria for acceptance. The percentage of single-end reads (%_Mapped_SE_reads) that can be mapped to the reference, out of the total number of single-end reads, is greater than 80 %. The percent of all unique total pairs that have both read-ends aligning to the same chromosome and have an insert size greater than or equal to 15 kb (percent long-cis (greater than 15 kb)) is greater than 30 % and the ratio of long-cis to Trans data (Lcis_trans_ratio) is greater than one. This ratio is the signal to noise ratio for translocation calling. HiC data QC statistics for the deeply sequenced Illumina data can be found in Supplementary File 2, Arima Deep QC.

## Usage Notes

We expect these extensive data sets to be useful for methods development for somatic variant calling, personalized tumor-normal analysis, and tumor genome assembly. As GIAB develops initial somatic benchmarks for batch 0823p23 of this tumor cell line, methods can be optimized and evaluated against these benchmarks. In the long-term, larger batches of cells from the tumor cell line can be characterized for differences in somatic mutations relative to batch 0823p23, enabling a more sustainable supply of DNA and cells. Future work will be needed to understand the genomic stability of the tumor cell line, but our initial limited data from different passages can be used to gain a preliminary understanding of differences.

### Code Availability

Scripts used for validation and figures are located on GitHub at https://github.com/usnistgov/giab-HG008-data-paper.

## Supplementary Material

Supplement 1

Supplement 2

Supplement 3

## Figures and Tables

**Table 1:** Batch genomic DNA (gDNA) isolation for distribution. DNA isolations from the three samples were performed for distribution to laboratories for measurement. Isolated DNA was sent to several sites for genome-scale measurements as described in Table 2. To confirm gDNA was fit for purpose, yield and length of gDNA was measured at different sites prior to use. Sizing and N50 reported are approximated values from three measurement sites. Genomic DNA yield from normal tissues for the separate isolations is noted in parentheses. Two sizing measurements are given for sites that measured sizing, prior to their measurements.

	Measurement Method	HG008-T	HG008-N-D	HG008-N-P
**Input**	NA	5 million non-viable tumor cells from batch 0823p23	two separate isolations of 10 mg of normal duodenal tissue were performed	two separate isolations of 10 mg of normal pancreatic tissue were performed
**Genomic DNA Yield**	Qubit	60 μg	34 μg (18 μg and 16 μg)	16 μg (5 μg and 11 μg)
**Read N50**	ONT sequencing	30 kb	10 kb	8 kb
**Sizing**	FemtoPulse	58 kb	NA	26 kb
	Tapestation	NA	53 kb	NA

**Table 2:** Summary of measurements made for HG008 tumor and normal samples. Sample fields denote the starting material received by the measurement institution; cells, tissue or isolated genomic DNA. Tumor samples derive from either a large, homogeneous batch of cells distributed in 2023 (0823p23) by the Liss lab at MGH, from a series of 2022 Liss lab passages starting with the p13 stock that batch 0823p23 was grown from or from NIST passages in 2024.

Measurement Type	Measurement Tecnology	Dataset ID	HG008-T	HG008-N-D	HG008-N-P
Bulk PCR-free short-read whole genome sequencing	Illumina NovaSeq 6000	ILMN-PCR-free-1	initial growth 2022, p36 cells		pancreatic tissue
		ILMN-PCR-free-2	0823p23 cells	duodenal tissue	
		ILMN-PCR-free-3	0823p23 cells	isolated DNA	
	Element Aviti short insert long insert	Element-short-2	0823p23 DNA	isolated DNA	isolated DNA
		Element-long-2	0823p23 DNA	isolated DNA	isolated DNA
	PacBio Onso	PB-Onso-1	0823p23 DNA	isolated DNA	
	Ultima UG100	Ultima-bulk-1	0823p23 DNA	isolated DNA	
Bulk Hi-C short-read whole genome sequencing	Phase Genomics CytoTerra and Arima HiC-Illumina NovaSeq 6000	HiC-ILMN-1	initial growth 2022, p38 viable cells		
		HiC-ILMN-2	0823p23 cells	duodenal tissue	
Bulk PCR-free long-read whole genome sequencing	PacBio Revio (HiFi)	PB-HiFi-1	0823p23 isolated DNA		isolated DNA
		PB-HIFi-2	0823p23 cells	duodenal tissue	
	Oxford Nanopore Technologies (ONT) PromethlON (standard - simplex)	ONT-std-1		duodenal tissue	pancreatic tissue
		ONT-std-2	0823p23 cells		
	ONT (Ultra Long)	ONT-UL-1	0823p23 cells		
Single-cell whole genome sequencing	BioSkryb single-cell Illumina WGS	sc-ILMN-1	0823p23 cells		
	Bioskryb single-cell Ultima WGS	sc-Ultima-1	0823p23 DNA		
Optical Mapping	Bionano Saphyr	Bionano-1	0823p23 cells		
Cytogenetic Analysis	G-banded Karyotyping	karyotpying-1	initial growth 2022, p31 viable cells		
		karyotpying-2	2024 NIST p18		
	Directional Genomic Hybridization	dGH-1	2024 NIST p21		

**Table 3:** Short-read Metrics. Summary of metrics generated by the GA4GH Quality Control pipeline, Samtools and Mosdepth for validation of short-read PCR-free data. Although an Element-short-1 dataset was generated, it is not presented, rather data from newer chemistry in the second dataset is provided, Element-short-2.

Dataset ID	GIAB sample ID	Technology	mismatch rate	average length (bp)	avg. insert size (bp)	Insert size SD	percent properly paired	mean autosome coverage	diploid mean coverage	haplold mean coverage	NRPCC
ILMN-PCR-free-1	HG008-N-P	Illumina NovaSeq 6000 WGS PCR-free 2×150	0.6	151	444	102	97.2	150			
	HG008-T		0.5	151	427	97	97.6	140	191	96	48
ILMN-PCR-free-2	HG008-N-D		0.5	151	417	94	96.6	169			
	HG008-T		0.6	151	432	97	96.8	141	195	98	49
ILMN-PCR-free-3	HG008-N-D		0.6	150	444	102	97.1	115			
	HG008-T		0.5	149	454	102	98.3	118	161	80	40
Element-short-2	HG008-N-D	Element Aviti short insert 2×150	0.4	147	383	144	94.7	72			
	HG008-N-P		0.4	147	379	161	87.1	70			
	HG008-T		0.4	148	404	147	89.5	70	102	51	26
Element-long-2	HG008-N-D	Element Aviti long insert 2×150	0.6	149	793	440	97.2	26			
	HG008-N-P		0.6	149	880	459	97.4	19			
	HG008-T		0.5	149	767	433	98.6	22	31	15	8
PB-Onso-1	HG008-N-D	PacBio Onso 2×150	0.4	143	321	103	96.6*	48			
	HG008-T		0.3	145	333	105	98*	100	136	68	34
Ultima-bulk-1	HG008-N-D	Ultima UG100 300bp SE	0.8	302	NA	NA	99.9	232			
	HG008-T		0.8	294	NA	NA	99.9	212	306	153	77

* The Onso percent properly paired metric presented represents pairing metric following filtering of 1 bp reads described with the Onso methods. Onso data presented as part of the HG008 dataset is not filtered.

**Table 4:** Long-read Metrics. Summary of metrics generated from Cramino and Mosdepth for validation of PacBio HiFi and ONT data.

Dataset ID	GIAB sample ID	Technology	number of alignments	percent from total reads	yield (Gb)	mean coverage	N50	median identity	diploid mean coverage	haploid mean coverage	NRPCC
PB-Hifi-1	HG008-N-P	PacBio HiFi	6,903,194	100	110	35	16,764	99.8			
	HG008-T		14,502,556	100	245	79	17,833	99.8	116	58	29
PB-Hifi-2	HG008-N-D		13,268,351	100	212	68	16,658	99.8			
	HG008-T		12,974,130	100	224	72	18,132	99.8	106	53	27
ONT-std-1	HG008-N-D	ONT-std	38,959,690	79.8	292	94	12,516	99.2			
	HG008-N-P		9,591,713	77.3	126	41	24,460	99.1			
ONT-std-2	HG008-T		6,673,103	80.2	138	45	35,497	99.2	63	31	16
ONT-UL-1	HG008-T	ONT-UL	2,576,733	79.1	110	39	118,692	98.7	54	27	14
