# Supplementary material for: Development and extensive sequencing of a broadly-consented Genome in a Bottle matched tumor-normal pair
Source: bioRxiv. 2025 Jun 14:2024.09.18.613544. Originally published 2024 Sep 22. Preprint. [Version 4] doi: 10.1101/2024.09.18.613544 (PMC11429686; doi:10.1101/2024.09.18.613544)
Supplement: Supplement 1 [file media-1.pdf]

Supplementary Information Tables

1. Table S1 Data records
2. Table S2 Validation tools
3. Table S3 QC metrics descriptions
4. Table S4 HG008 STR typing

Measurement Technology	Measurement Institution	Dataset ID	Sample Source	Sample Material Received	File Type(s)	Number of files	Data Access
Illumina NovaSeq 6000 PCR-Free WGS	Baylor College of Medicine, Human Genome Sequencing Center	ILMN-PCR-free-1	normal pancreatic tissue	tissue	paired-end reads (R1/R2) in fastq.gz format	x2 (fastq.gz, R1/R2)	SRA
			PDAC tumor cell line	initial growth 2022, p36 cells		x2 (fastq.gz, R1/R2)	
		ILMN-PCR-free-2	normal duodenal tissue	tissue		x2 (fastq.gz, R1/R2)	
			PDAC tumor cell line	batch 0823p23 cells		x8 (fastq.gz, R1/R2)	
	New York Genome Center	ILMN-PCR-free-3	normal duodenal tissue	isolated DNA		x24 (fastq.gz, R1/R2)	
			PDAC tumor cell line	batch 0823p23 cells		x24 (fastq.gz, R1/R2)	
Element Aviti	Element Biosciences	Element-short-3	normal duodenal tissue	isolated DNA	paired-end reads (R1/R2) in fastq.gz format	x2 (fastq.gz, R1/R2)	
			normal pancreatic tissue	isolated DNA		x2 (fastq.gz, R1/R2)	
			PDAC tumor cell line	batch 0823p23 isolated DNA		x2 (fastq.gz, R1/R2)	
		Element-long-1	normal duodenal tissue	isolated DNA		x2 (fastq.gz, R1/R2)	
			normal pancreatic tissue	isolated DNA		x2 (fastq.gz, R1/R2)	
			PDAC tumor cell line	batch 0823p23 isolated DNA		x2 (fastq.gz, R1/R2)	
PacBio Onso	PacBio	PB-Onso-1	normal duodenal tissue	isolated DNA	paired-end reads (R1/R2) in fastq.gz format	x2 (fastq.gz, R1/R2)	
			PDAC tumor cell line	batch 0823p23 isolated DNA		x2 (fastq.gz, R1/R2)	
Ultima UG100	Ultima Genomics	Ultima-bulk-1	normal duodenal tissue	isolated DNA	aligned reads in .cram format with associated .cram.crai index	x2 (.cram/.crai)	
			PDAC tumor cell line	batch 0823p23 isolated DNA		x2 (.cram/.crai)	
		Ultima-ppmSeq-2	normal duodenal tissue	isolated DNA		x2 (.cram/.crai)	
			PDAC tumor cell line	batch 0823p23 isolated DNA		x2 (.cram/.crai)	
HiC-Illumina	Phase Genomics	HiC-ILMN-1	PDAC tumor cell line	initial growth 2022, p38 viable cells	paired-end reads (R1/R2) in fastq.gz format	x4 (fastq.gz, R1/R2)	
	Arima Genomics - Baylor College of Medicine	HiC-ILMN-2	normal duodenal tissue	tissue		x2 (fastq.gz, R1/R2)	
			PDAC tumor cell line	batch 0823p23 cells		x2 (fastq.gz, R1/R2)	
	Phase Genomics	HiC-ILMN-3	PDAC tumor cell line	2024 NIST p21 cells		x2 (fastq.gz, R1/R2)	
	Dovetail Genomics	HiC-ILMN-4	PDAC tumor cell line	2024 NIST p21 cells		x8 (fastq.gz, R1/R2)	
PacBio Revio (HiFi)	PacBio	PB-HiFi-1	normal pancreatic tissue	isolated DNA	raw reads in unaligned .bam format	x1 (.bam)	
			PDAC tumor cell line	batch 0823p23 isolated DNA		x2 (.bam)	
	Baylor College of Medicine, Human Genome Sequencing Center	PB-HiFi-2	normal duodenal tissue	tissue		x2 (.bam)	
			PDAC tumor cell line	batch 0823p23 cells		x2 (.bam)	
Oxford Nanopore Technologies (ONT) PromethION (standard - simplex)	Northeastern University, Genome Technology Lab	ONT-std-1	normal duodenal tissue	tissue	raw reads in unaligned .bam format	x4 (.bam)	
			normal pancreatic tissue	tissue		x3 (.bam)	
	University of CA Santa Cruz Genomics Institute	ONT-std-2	PDAC tumor cell line	batch 0823p23 cells		x1 (.bam)	
Oxford Nanopore Technologies (ONT) PromethION (Ultra Long)	University of CA Santa Cruz, Genomics Institute	ONT-UL-1	PDAC tumor cell line	batch 0823p23 cells		x3 (.bam)	
	Northeastern University, Genome Technology Lab	ONT-UL-2	PDAC tumor cell line	batch 0823p23 cells		x1 (.bam)	
Single-Cell (BioSkryb ResolveDNA) WGS Illumina	BioSkryb Genomics	sc-ILMN-1	PDAC tumor cell line	batch 0823p23 cells	paired-end reads (R1/R2) in fastq.gz format	x240 (.fastq.gz, R1/R2)	
Single-Cell (BioSkryb ResolveDNA) WGS Ultima	BioSkryb - Ultima Genomics	sc-Ultima-1	PDAC tumor cell line	Converted sc-ILMN-1 libraries to Ultima libraries	aligned (GRCh38) reads in . cram format with associated . cram.crai index	x238 (.cram./.crai)	
Saphyr Optical Mapping	Bionano Genomics	Bionano-1	PDAC tumor cell line	batch 0823p23 cells	raw data view of molecule and label information in .bnx format and description of SVs detected by two genome maps in .smap format	x3 (.bnx/.smap/.cmap)	NCBI supplementary
Karyotyping	KaryoLogic	karyotpying-1	PDAC tumor cell line	initial growth 2022, p31 viable cells	karyogram provided in cytogenetic analysis report (.pdf)	x2 (.pdf/.xlsx)	figshare
		karyotpying-2	PDAC tumor cell line	2024 NIST p18 cells		x1 (.pdf)	
Directional Genomic Hybridization (dGH) KROMASURE Screen	KROMATID	dGH-1	PDAC tumor cell line	2024 NIST p21 cells	karyogram provided as part of Figure 2	NA	karyogram provided as part of Figure 2

Table S1 Dataset descriptions

Summary of HG008 datasets, including measurement institution, file types, and data access information. The sample material received denotes the starting material received by the measurement institution; cells, tissue or isolated genomic DNA. Tumor samples derive from either a large, homogeneous batch of cells distributed in 2023 (0823p23) by the Liss lab at MGH, from a series of 2022 Liss lab passages starting with the p13 stockthat batch 0823p23 was grown from or from NIST passages in 2024 started from Liss lab p13 cryopreserved cells.

Dataset ID	Mapping Tools		Reference Genome	Validation Tools	
ILMN-PCR-free-1	BWA-MEM	BWA: https://github.com/lh3/bwa	GRCh38-GIABv3: https://ftp-trace.ncbi.nlm.nih.gov/giab/ftp/release/references/GRCh38/GRCh38_GIABv3_no_alt_analysis_set_maskedGRC_decoys_MAP2K3_KMT2C_KCNJ18.fasta.gz	FastQC<br>GA4GH QC pipeline<br>Mosdepth (run as part of snakemake workflow)	FastQC: https://github.com/s-andrews/FastQC<br>GA4GH Pipeline: https://github.com/c-BIG/NPM-sample-qc/tree/v0.11.0/<br>Mosdepth Snakemake wrapper: https://snakemake-wrappers.readthedocs.io/en/stable/wrappers/mosdepth.html
ILMN-PCR-free-2					
Element-short-3					
Element-long-1					
PB-Onso-1					
ILMN-PCR-free-3		NYGC somatic pipeline v7: https://bitbucket.nygenome.org/projects/WDL/repos/somatic_dna_wdl/browse/README_pipeline.md?at=refs%2Fheads%2Fgiab#deliverables			
Ultima-bulk-1	Optimized version of BWA (UA) for Ultima data	Ultima Aligner: https://hub.docker.com/r/ultimagenomics/alignment		GA4GH QC pipeline<br>Mosdepth (run as part of snakemake workflow)	GA4GH Pipeline: https://github.com/c-BIG/NPM-sample-qc/tree/v0.11.0/<br>Mosdepth Snakemake wrapper: https://snakemake-wrappers.readthedocs.io/en/stable/wrappers/mosdepth.html
HiC-ILMN-1	BWA-MEM (as part of the HiC alignment and QC protocol)	HiC alignment and QC: https://phasegenomics.github.io/2019/09/19/hic-alignment-and-qc.html	GRCh38.p13: https://www.ncbi.nlm.nih.gov/datasets/genome/GCF_000001405.39/	hic_qc (as part of the HiC alignment and QC protocol)	HiC QC script: https://github.com/phasegenomics/hic_qc
HiC-ILMN-3					
HiC-ILMN-2	HiCUP (as part of the Arima SV pipeline)	Arima SV pipeline: https://arimagenomics.com/wp-content/files/Bioinformatics-User-Guide-Arima-Structural-Variant-Pipeline.pdf	hg38: no path provided	QC part of the Arima SV pipeline	Arima SV pipeline: https://arimagenomics.com/wp-content/files/Bioinformatics-User-Guide-Arima-Structural-Variant-Pipeline.pdf
HiC-ILMN-4	BWA-MEM (as part of the Alignment and Proximity-Ligation QC)	Alignment and Proximity-Ligation QC: https://varilink.readthedocs.io/en/latest/library_qc.html	hg38: https://hgdownload.soe.ucsc.edu/goldenPath/hg38/bigZips/hg38.fa.gz	QC output from pairtools (as part of the Alignment and Proximity-Ligation QC)	pairtools: https://github.com/open2c/pairtools
PB-HiFi-1	pbmm2 (as part of PacBio WDL germline (v1.1.1) or somatic (v0.7) pipelines)	pbmm2: https://github.com/PacificBiosciences/pbmm2<br>PacBio germline pipeline v1.1.1: https://github.com/PacificBiosciences/HiFi-human-WGS-WDLPacBio<br>somatic pipeline v0.7: https://github.com/PacificBiosciences/HiFi-Somatic-WDL	GRCh38-GIABv3: https://ftp-trace.ncbi.nlm.nih.gov/giab/ftp/release/references/GRCh38/GRCh38_GIABv3_no_alt_analysis_set_maskedGRC_decoys_MAP2K3_KMT2C_KCNJ18.fasta.gz	Cramino<br>Nanoplot<br>Mosdepth<br>Samtools stats	Cramino: https://github.com/wdecoster/cramino<br>Nanoplot: https://github.com/wdecoster/NanoPlot<br>Mosdepth Snakemake wrapper: https://snakemake-wrappers.readthedocs.io/en/stable/wrappers/mosdepth.html<br>Samtools: https://github.com/samtools/samtools
PB-HiFi-2					
ONT-std-1	Samtools<br>Minimap2	Samtools: https://github.com/samtools/samtools<br>minimap2: https://github.com/lh3/minimap2		PycoQC<br>Cramino<br>Nanoplot<br>Mosdepth<br>Samtools stats	pycoQC: https://github.com/a-slide/pycoQC<br>Cramino: https://github.com/wdecoster/cramino<br>Nanoplot: https://github.com/wdecoster/NanoPlot<br>Mosdepth Snakemake wrapper: https://snakemake-wrappers.readthedocs.io/en/stable/wrappers/mosdepth.html<br>Samtools: https://github.com/samtools/samtools
ONT-std-2					
ONT-UL-1					
ONT-UL-2					
sc-ILMN-1	Sention BWA-MEM (as part of BJ-DNA-QC pipeline)	BJ-DNA-QC pipeline: https://docs.basejumper.bioskryb.com/pipelines/secondary/bj-dna-qc/1.8.2/docs/	GRCh38: * gs://gcp-public-data--broad-references/hg38/v0/Homo_sapiens_assembly38.fasta	validation as part of the BJ-DNA-QC pipeline	BJ-DNA-QC pipeline: https://docs.basejumper.bioskryb.com/pipelines/secondary/bj-dna-qc/1.8.2/docs/
sc-Ultima-1	Optimized version of BWA (UA) for Ultima data	Ultima Aligner: https://hub.docker.com/r/ultimagenomics/alignment	GRCh38: * gs://gcp-public-data--broad-references/hg38/v0/Homo_sapiens_assembly38.fasta	Ultima custom UG100 tool	Ultima Sorter: https://hub.docker.com/r/ultimagenomics/sorter
Ultima-ppmSeq-1	Optimized version of BWA (UA) for Ultima data	Trim, align and sort using ppmSeqPreprocess workflow: https://github.com/Ultimagen/healthomics-workflows/tree/0c827f223b47d84736cea7626af6ddb8ca0a90a0/workflows/ppmSeq_preprocess	GRCh38: * gs://gcp-public-data--broad-references/hg38/v0/Homo_sapiens_assembly38.fasta	GA4GH QC pipeline	GA4GH pipeline: https://github.com/c-BIG/NPM-sample-qc/tree/v0.11.0/
Bionano-1	Bionano Rare Variant Analysis pipeline (included in Bionano Access software)	Bionano proprietary tool	GRCh38: https://ftp.ncbi.nlm.nih.gov/genomes/all/GCA/000/001/405/GCA_000001405.15_GRCh38/seqs_for_alignment_pipelines.ucsc_ids/GCA_000001405.15_GRCh38_no_alt_analysis_set.fna.gz	Bionano Access version 1.8	Bionano proprietary tool

Table S2 Validation Tools

Summary of tools used for alignment and validation.

*Information for accessing and use of files from the Broad Resoruce bundle can be found at https://gatk.broadinstitute.org/hc/en-us/articles/360035890811-Resource-bundle

Tool	Metric	Description
Cramino	number of alignments	Total number of reads aligned to the reference genome.
	percent from total reads	Percentage of reads successfully aligned.
	yield (Gb)	The total amount of sequencing data generated, measured in gigabases (Gb).
	mean coverage	The average number of times each base in the genome is read during sequencing.
	N50	Half of the sequenced bases are in reads longer than the N50.
	median identity to GRCh38	Median fraction (%) of read bases matching the GRCh38 reference genome.
Samtools stats	samtools error rate (%)	The proportion (%) of bases in the reads that do not match the reference. The output of samtools stats is commonly referred to as the error rate. The samtools error rate measures substitution and indel differences between the reads and the reference, including differences resulting from true variants.
	average length (bp)	The average length of the reads in the dataset.
	avg. insert size (bp)	The average length of the DNA fragments that are sequenced. The output of the samtools stats is commonly referred to as the insert size average.
	insert size SD	The variability in the insert sizes. The output of the samtools stats is commonly referred to as the insert size standard deviation.
	properly paired (%)	The percentage of read pairs that are aligned with the expected pairing. The output of the samtools stats is commonly referred to as the percentage of properly paired reads.
	mapped reads (%)	The percentage of reads mapping to the reference genome. This is calculated using outputs from samtools stats ("reads mapped" / "raw total sequences" x 100).
	bases mapped (%)	The percentage of bases, ignoring clipping, that map to the reference genome. This is calculated using outputs from samtools stats, (bases mapped (cigar))/(total length) x 100).
GA4GH mosdepth	mean autosome coverage	The mean sequencing coverage derived from high-quality, non duplicated reads, primary alignments in the autosome non-gap regions of the reference used.
Mosdepth	haploid mean coverage	Estimated coverage of haploid regions assuming no whole genome doubling, derived from the mean sequencing coverage derived from high-quality, non duplicated reads, primary alignments in chromosome 4 for HG008-T, which is haploid.
	diploid mean coverage	Estimated coverage of diploid regions assuming no whole genome doubling, calculated as double the haploid mean coverage.
	NRPCC	Estimated coverage per haplotype assuming whole genome doubling has occurred.
BioSkryb BJ-DNA-QC pipeline metrics	Pre-Seq Count	Estimation of the genomic coverage of a given library if it were to be run at the WGS level. Traditionally, BioSkryb Genomics has utilized the NIST cell line NA12878,in this cell line, which has no major genomic alterations, pre-seq counts of high-quality cells are typically greater than 3,000,000,000.
	% ChrM	Percentage of mitochondrial reads in the sequencing, defined as <2% for passing cells.
	% Chimera	Percentage of chimeric reads seen at the QC Sequencing level, defined as <20% for passing cells.
	Gini coefficient	The Gini coefficient assess assay and sequencing performance by measuring the diversity of reads sequenced, diversity of reads is reflective of homogeneity of coverage, the higher the homogeneity of coverage the greater the diversity of reads sequences. This is also typically associated with the ability to assess copy number variation at the QC sequencing level. Passing cells are below 0.1
Phase Genomics CytoTerra QC metrics	High-Quality	Refers to read pairs having MAPQ>0, are not PCR duplicates and map to different contigs or are >10kb apart.
	Same strand high quality read pairs	This statistic measures the percentage of high quality read pairs where both reads map to the same strand in the reference genome. This is indicative that the read is the result of a proximity ligation event which changes the orientation of the sequences relative to each other. Doubling this value gives an estimate of the total percentage of proximity ligation junctions present in the library. (15% minimum value acceptable)
	Fraction of high quality read pairs >10 kb apart	CytoTerra library success is dependent on the fraction of reads that contain long-range contact information. This stat measures the percentage of high quality read pairs that map >10 kb apart in the reference genome. (20% minimum acceptable value)
	Duplicate Reads	This measures the rate of PCR duplicate fragments present in the library and fits a saturation model to extrapolate the duplication rate at 100M read pairs. This is a critical measure of the complexity of a library. (40% maximum acceptable value).
	Intercontig read pairs	This measures the frequency which pairs of reads map to different contigs in an assembly. Since in the human genome, contigs are (nearly) complete chromosomes, intercontig read pairs is an estimate of spurious proximity ligation events. There are exceptions to this threshold in highly rearranged genomes since you then expect interchromosomal read pairs due to translocations. (30% maximum acceptable value)

Table S3 Validation metrics descriptions

Overview of tools and description of their output metrics presented as part of validation for the HG008 datasets.

Normal Duodenal Tissue	
AMEL	X
D3S1358	14, 15
D1S1656	16, 17.3
D2S441	11
D10S1248	14
D13S317	11, 12
Penta E	12, 18
D16S539	11
D18S51	10, 15
D2S1338	17, 18
CSF1PO	10, 11
Penta D	12
TH01	8, 9.3
vWA	14, 18
D21S11	28, 29
D7S820	7, 8
D5S818	11
TPOX	10, 11
D8S1179	10, 13
D12S391	17, 18
D19S433	12, 13
SE33	18, 23
D22S1045	11, 15
FGA	21, 25

Normal Pancreatic Tissue	
AMEL	X
D3S1358	14, 15
D1S1656	16, 17.3
D2S441	11
D10S1248	14
D13S317	11, 12
Penta E	12, 18
D16S539	11
D18S51	10, 15
D2S1338	17, 18
CSF1PO	10, 11
Penta D	12
TH01	8, 9.3
vWA	14, 18
D21S11	28, 29
D7S820	7, 8
D5S818	11
TPOX	10, 11
D8S1179	10, 13
D12S391	17, 18
D19S433	12, 13
SE33	18, 23
D22S1045	11, 15
FGA	21, 25

HG008-T cell line (batch 0823p23)	
AMEL	X
D3S1358	15
D1S1656	16, 17.3
D2S441	11
D10S1248	14
D13S317	11, 12
Penta E	12, 18
D16S539	11
D18S51	10
D2S1338	17, 18
CSF1PO	11
Penta D	12
TH01	8, 9.3
vWA	14, 18
D21S11	28
D7S820	8
D5S818	11
TPOX	10, 11
D8S1179	10, 13
D12S391	17, 18
D19S433	12
SE33	23
D22S1045	15
FGA	25

HG008-T cell line (NIST passage 21)	
AMEL	X
D3S1358	15
D1S1656	16, 17.3
D2S441	11
D10S1248	14
D13S317	11, 12
Penta E	12, 18
D16S539	11
D18S51	10
D2S1338	17, 18
CSF1PO	11
Penta D	12
TH01	8, 9.3
vWA	14, 18
D21S11	28
D7S820	8
D5S818	11
TPOX	10, 11
D8S1179	10, 13
D12S391	17, 18
D19S433	12
SE33	23
D22S1045	15
FGA	25

Table S4 STR genotyping

STR genotyping was conducted on DNA isolated from normal pancreatic and duodenal tissues, as well as the HG008-T cells (batch 0823p23 and NIST passage 21). The resulting allelic profiles for each marker are presented in the accompanying table. Notably, some markers in the HG008-T samples exhibited allelic loss due to large deletions previously identified in other analyses, as discussed in the Technical Validation section.
