# Supplementary material for: Development and extensive sequencing of a broadly-consented Genome in a Bottle matched tumor-normal pair
Source: bioRxiv. 2025 Jun 14:2024.09.18.613544. Originally published 2024 Sep 22. Preprint. [Version 4] doi: 10.1101/2024.09.18.613544 (PMC11429686; doi:10.1101/2024.09.18.613544)
Supplement: Supplement 2 [file media-2.pdf]

UG ppmSeq™ Genomic DNA Library Preparation Protocol

Optimized with NEBNext UltraShear™, NEBNext® Ultra™ II End Repair/dA-Tailing Module, NEBNext® Ultra™ II Ligation Module

Rev. 01

UG ppmSeq™ Genomic DNA Library Preparation Protocol
D1000993 Rev. 01 | 30 September 2024

© 2024, Ultima Genomics, Inc. All rights reserved.

UG 100™, ppmSeq™, ULTIMA GENOMICS®, and the Ultima Genomics logo are trademarks of Ultima Genomics, Inc. Other product names and trademarks are the property of their respective owners.

Table of Contents

1. **Revision History** **1**
2. **Document Conventions** **2**
   Icons and Text Conventions 2
3. **Purpose** **3**
4. **Support** **4**
5. **Workflow Overview** **5**
6. **Materials Required** **6**
   Reagents Supplied by Ultima Genomics 6
   Indexed UG ppmSeq™ v3 Adapters 6
   Customer Supplied Reagents 6
   Additional Reagents 7
   Customer Supplied Consumables 7
   Recommended Equipment 7
7. **Procedure** **8**
   Fragment gDNA Using NEBNext UltraShear 8
   End Repair and A-Tailing Using NEBNext Ultra II End Repair/dA-Tailing Module 9
   Adapter Ligation with NEBNext Ultra II Ligation Module 9
   Library Clean-Up 10
   Size Selection 11
8. **Library QC** **12**
9. **Best Practices** **13**
   General Dilution Tips 13
   Dilution Guidelines and Factors for Library Preparation 13
10. **Safety and Regulatory** **14**
   General 14
   Waste 14
   Safety Data Sheets (SDS) 14
   Protocol Use 14

1. Revision History

Revision	Release Date	Description of Changes
01	10/30/2024	Initial release

2. Document Conventions

The following conventions are used in this document.

Icons and Text Conventions

Icon	Description
	**NOTE**<br>Alerts you of important information about a system feature or process.
	**CAUTION!**<br>Alerts you of a hazardous situation that could result in potential loss of data or damage to the system.
	**WARNING!**<br>Alerts you of a hazardous situation that could result in potential personal injury.
	**SAFE STOPPING POINT!**<br>Indicates a safe stopping point in a procedure.
Hyperlink	Hyperlinks are highlighted in color and are underlined. Click on the hyperlink to go or open the corresponding location (for example: another section in the document, a website, or an e-mail address).
Boldface	Button labels are highlighted in boldface. For example, Start button.

3. Purpose

This protocol generates ppmSeq™ libraries from genomic DNA (gDNA) using NEBNext UltraShear™, NEBNext® Ultra™ II End Repair/dA-Tailing, and NEBNext® Ultra™ II Ligation reagents. The resulting PCR-free libraries are compatible with the Ultima Genomics® UG 100™ Sequencer.

This protocol was developed based on the NEBNext UltraShear™ with NEBNext® Ultra™ II End Repair/dA-Tailing Module and NEBNext® Ultra™ II Ligation Module protocols with steps added or modified for sequencing compatibility on the UG 100™ Sequencing Platform.

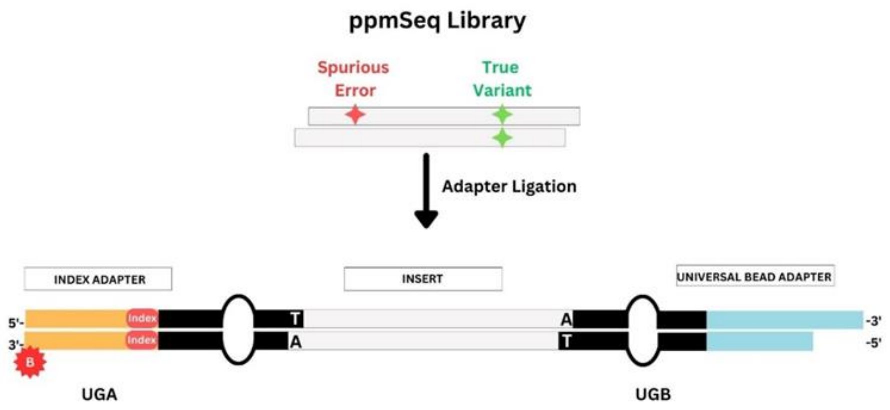

Figure 1: ppmSeq™ Library Workflow. This schematic illustrates the ppmSeq™ Library Workflow, designed for genomic DNA samples.

4. Support

Contact support@ultimagen.com for support and assistance.

Mailing Address:

Ultima Genomics, Inc.
4209 Technology Drive
Fremont, CA 94538
USA

5. Workflow Overview

ppmSeq™ Genomic DNA Library Preparation (16 Samples)	Approximate Time
**Enzymatic DNA Fragmentation**	
Add buffer and enzyme mixes to sample	**60 mins**
Run thermocycler	
**End Repair and dA-Tailing**	
Add buffer and enzyme mixes to sample	**75 mins**
Run thermocycler	
**Adapter Ligation**	
Add adapters to sample	
Add ligation master mix to sample	**80 mins**
Incubate at 25°C for 60 mins	
**Cleanup and Size-Selection**	
**SPRI Cleanup**	
Proceed with SPRI cleanup (0.79x).	
Transfer the supernatant to a new tube after the first SPRI cleanup stage	**90 mins**
**SPRI Size-Selection**	
Proceed with SPRI size-selection (0.65x/1x).	
Elute the size-selected library	
**Sample QC**	
Bioanalyzer, TapeStation, or Fragment Analyzer	**150 mins**
qPCR	
**Total Time**	**~7 hour 35 mins**

Detailed breakdown of workflow steps, from input to quality control (QC), with estimated time and hands-on duration for 16 samples.

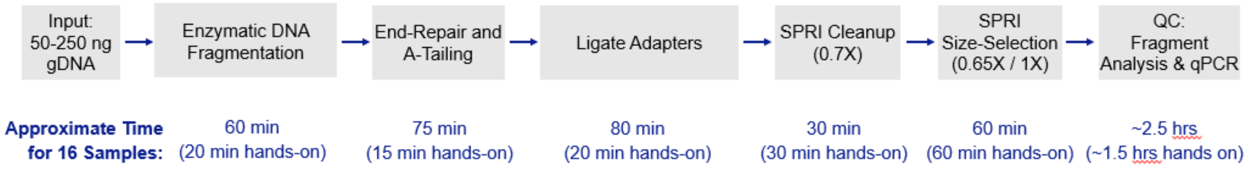

6. Materials Required

Reagents Supplied by Ultima Genomics

Indexed UG ppmSeq™ v3 Adapters

Reagent Name	Part Number	Storage	Reaction Size
UG ppmSeq™ Adapter Plate v3 (96 Barcodes)	L21	-20°C	192 rxns per plate
UG ppmSeq™ Adapter v3 Tubes (16 Barcodes)	500, 501, 502, 503, 504, 505, 506, 507, 508, 509, 510, 511, 512, 513, 514, 515	-20°C	8 rxns per tube

For ordering inquiries related to Indexed UG ppmSeq™ v3 Adapters, please contact Ultima Genomics or reach out to your Field Application Scientist (FAS) from Ultima Genomics.

Customer Supplied Reagents

NEBNext UltraShear™ (catalog #M7634)

Included in the kit (information according to manufacturer):

Contents	Storage
NEBNext UltraShear™	-20°C
NEBNext UltraShear™ Reaction Buffer	
500 mM DTT	

NEBNext® Ultra™ II End Repair/dA-Tailing Module (catalog #E7546)

Included in the kit (information according to manufacturer):

Contents	Storage
NEBNext®Ultra™ II End Prep Enzyme Mix	-20°C
NEBNext®Ultra™ II End Prep Reaction Buffer	

NEBNext® Ultra™ II Ligation Module (catalog #E7595)

Included in the kit (information according to manufacturer):

Contents	Storage
NEBNext®Ultra™ II Ligation Enhancer	-20°C
NEBNext®Ultra™ II Ligation Master Mix	

Alternatively, NEBNext® Ultra™ II DNA PCR-free Library Prep Kit for Illumina (catalog #E7410) can be purchased in lieu of the NEBNext® Ultra™ II End Repair/dA-Tailing Module (catalog #E7546) and NEBNext® Ultra™ II Ligation Module (catalog #E7595).

NEBNext® Library Quantification Kit for UG Libraries: For ordering inquiries related to the NEBNext® Library Quantification Kit for UG Libraries, please contact your Field Application Scientist (FAS) from Ultima Genomics.

Additional Reagents

Item	Source	Part Number
Agencourt™ AMPure™ xP Kit (or similar SPRI beads)	Beckman Coulter	A63880
Agilent™ High Sensitivity DNA Kit or Agilent Genomic DNA ScreenTape	Agilent	5067-4626 (or similar)
Ethanol, Absolute (200 Proof), Molecular Biology Grade	Fisher Scientific	BP2818100
Buffer EB (10mM Tris-HCl, pH 8.5)	Qiagen	19086
1 x TE (10 mM Tris pH 8.0, 1 mM EDTA)	Fisher Scientific	BP2473500

Customer Supplied Consumables

Item	Source	Part Number
Barrier Pipette Tips	Rainin	LTS Pipette Tips
0.2 mL PCR Tubes or Plates	USA Scientific	1402-2700, 1402-9100 or similar
Eppendorf LoBind™ Tubes, 1.5 mL	Eppendorf	22431021

Recommended Equipment

Item	Source	Part Number
Pipettors 1–1000 µL	Rainin	17014496, 30386739
Magnetic Rack (for 0.2 mL tubes)	NEB	S1515S, or Thermo Scientific 492025
Vortex Mixer, Vortex-Genie® 2	USA Scientific	7404-5600
Microcentrifuge	USA Scientific	2621-0016
Agilent™ 2100 Bioanalyzer™ Instrument or Agilent™ 4150 TapeStation System	Agilent	G2939AA or G2992AA
Thermalcycler	BioRad	Heated lid, 0.2 µL tube (or similar)
Real-Time PCR (qPCR) Instrument	User-supplied	User-supplied

7. Procedure

Fragment gDNA Using NEBNext UltraShear

1. For each sample, use 50-250 ng of purified genomic DNA (gDNA) diluted to a final volume of 26 µL in 1x TE (10 mM Tris pH 8.0, 1 mM EDTA).

NOTE

The fragmentation reaction is sensitive to the buffer composition, and poorly optimized fragmentation can result in low library yields. Only 1x TE (10 mM Tris pH 8.0, 1 mM EDTA) should be used to dilute the gDNA. If possible, the gDNA should be purified directly into 1x TE. If the purified gDNA is in another buffer and a significant volume will be added to the fragmentation reaction, it is advised that the gDNA be buffer exchanged into 1x TE. Furthermore, contaminants from certain gDNA extraction techniques can inhibit fragmentation. Performing a cleanup on the gDNA should rescue fragmentation.

2. Thaw NEBNext UltraShear™ Reaction Buffer completely, then vortex quickly to mix. Place on ice until use.
3. Vortex the NEBNext UltraShear enzyme for 5-10 seconds before use, and place on ice.
4. Program the thermocycler with the following conditions. Set the temperature of the heated lid to 75°C. Start the program to pre-chill the thermocycler.

Step	Temp	Time
1	4°C	HOLD
2	45°C	25 min
3	65°C	15 min
4	4°C	Hold

NOTE

If library yield is low, it may be necessary to optimize the fragmentation time at 45°C. Aim to maximize the amount of fragmented gDNA in the 250-270 bp size range.

5. Prepare the fragmentation reactions in 0.2 mL PCR tubes or plates on ice:

Component	Volume Per Sample (µL)
gDNA sample	26
NEBNext UltraShear Reaction Buffer	14
NEBNext UltraShear	4
**Total Volume**	**44**

6. Vortex the reactions for 5-10 seconds, and spin briefly in a microcentrifuge.
7. Place the samples in the pre-chilled thermocycler and proceed with steps 2-4 of the thermocycler program.

SAFE STOPPING POINT!

This is a safe stopping point. Libraries can be stored overnight at -20°C.

End Repair and A-Tailing Using NEBNext Ultra II End Repair/dA-Tailing Module

NOTE
Only the NEBNext Ultra II End Prep Enzyme Mix and 500 mM DTT are used in this protocol. The NEBNext End Prep Reaction Buffer is not used.

1. Thaw the 500 mM DTT, then vortex to mix.
2. Prepare the End Repair and A-Tailing reactions on ice:

Component	Volume Per Sample (µL)
Fragmented gDNA	44
500 mM DTT	2
NEBNext Ultra II End Prep Enzyme Mix	3
**Total Volume**	**49**

3. Set a pipette to 40 µL, then pipette the samples up and down at least 10 times to mix. Spin briefly in a microcentrifuge.

NOTE
Thorough mixing is critical for optimal performance. The presence of a small amount of bubbles will not compromise performance.

4. Place the samples in a thermocycler with the heated lid set to ≥75°C, and run the following program:

Step	Temp	Time
1	20°C	30 min
2	65°C	30 min
3	4°C	Hold

5. Proceed immediately to Adapter Ligation.

Adapter Ligation with NEBNext Ultra II Ligation Module

1. Thaw the UG ppmSeq™ Adapter Plate v3. Vortex to mix, and spin briefly in a microcentrifuge.
2. Mix the NEBNext Ultra II Ligation Master Mix by pipetting up and down several times before use.
3. Prepare Ligation reactions:

Component	Volume Per Sample (µL)
End-Repaired and A-Tailed sample	49
Elution Buffer (10 mM Tris pH 8.0)	7.25
UG ppmSeq™ v3 Adapters (Indexed)	6.25
NEBNext Ultra II Ligation Master Mix	30

Component	Volume Per Sample (µL)
NEBNext Ligation Enhancer	1
**Total Volume**	**93.5**

NOTE
The Ligation Master Mix and Ligation Enhancer can be mixed ahead of time. This premix is stable for at least 8 hours at 4°C. Do not add the adapters to the premix.

4. Set a pipette to 80 µL, then pipette the samples up and down at least 10 times to mix. Spin briefly in a microcentrifuge.
5. Place the samples in a thermocycler with the heated lid turned off, and run the following program:

Step	Temp	Time
1	20°C	60 min
2	4°C	Hold

6. Proceed immediately to Library Clean-Up.

Library Clean-Up

1. Allow AMPure beads to warm to room temperature. Vortex to resuspend thoroughly.
2. Add 65.5 µL of AMPure beads (0.7 x final) to each ligation reaction.
3. Vortex the reactions for 10 seconds, then incubate for 5 minutes at room temperature.
4. Spin down, and place samples on magnet for 4 minutes.
5. Remove and discard supernatant.
6. Keeping samples on the magnet, add 200 µL of 80% ethanol. Incubate at room temperature for 30 seconds.
7. Remove and discard the ethanol supernatant.
8. Repeat wash: Keeping samples on the magnet, add 200 µL of 80% ethanol. Incubate at room temperature for 30 seconds.
9. Remove and discard the ethanol supernatant.
10. Spin down, place samples back on the magnet, and remove remaining ethanol. Air dry for 1 minute on magnet at room temperature.
11. Add 51 µL of Elution Buffer (10 mM Tris pH 8.0) to each sample.
12. Vortex 30 seconds, then incubate for 2 minutes at room temperature.
13. Spin down, and place samples on a magnet for 4 minutes.

NOTE
Do NOT discard supernatant!

14. Transfer the clear supernatant (50 µL) to a new tube, and discard beads.
15. Proceed to Size Selection or store overnight at -20°C.

SAFE STOPPING POINT!
This is a safe stopping point. Libraries can be stored overnight at -20°C.

Size Selection

1. Add 32.5 µL of AMPure beads to each sample (0.65 x final).
2. Vortex 30 seconds, then incubate samples for 5 minutes at room temperature.
3. Spin down, and place samples on a magnet for 4 minutes.

NOTE
Do NOT discard supernatant!

4. Transfer the supernatant (82.5 µL) to a new PCR tube, and discard beads.
5. Add 17.5 µL AMPure beads (1 x final) to the transferred supernatant.
6. Vortex 10 seconds, then incubate samples 5 minutes at room temperature.
7. Spin down, and place samples on a magnet for 4 minutes.
8. Remove and discard supernatant.
9. Keeping samples on the magnet, add 200 µL of 80% ethanol. Incubate at room temperature for 30 seconds.
10. Remove and discard the ethanol supernatant.
11. Repeat wash: Keeping the samples on the magnet, add 200 µL of 80% ethanol. Incubate at room temperature for 30 seconds.
12. Remove and discard the ethanol supernatant.
13. Spin down, and place samples back on the magnet.
14. Remove and discard the remaining ethanol. Air dry for 1 minute on the magnet at room temperature.
15. Add 63 µL Elution Buffer (10 mM Tris pH 8.0) to each sample.
16. Vortex for 30 seconds, then incubate samples for 2 minutes at room temperature.
17. Spin down, and place samples on a magnet for 2 minutes.

NOTE
Do NOT discard supernatant!

18. Transfer supernatant (63 µL) to a new tube, and discard beads.
19. Proceed to QC analysis (BioAnalyzer or TapeStation to determine mean fragment length, and qPCR to determine concentration)

SAFE STOPPING POINT!

This is a safe stopping point. Libraries can be stored for up to 1 week at 4°C or for 3 months at -20°C.

8. Library QC

1. Run 1µL of each library on a Bioanalyzer High Sensitivity Chip (or similar) to determine the mean library length in the range of 110-700 bp.

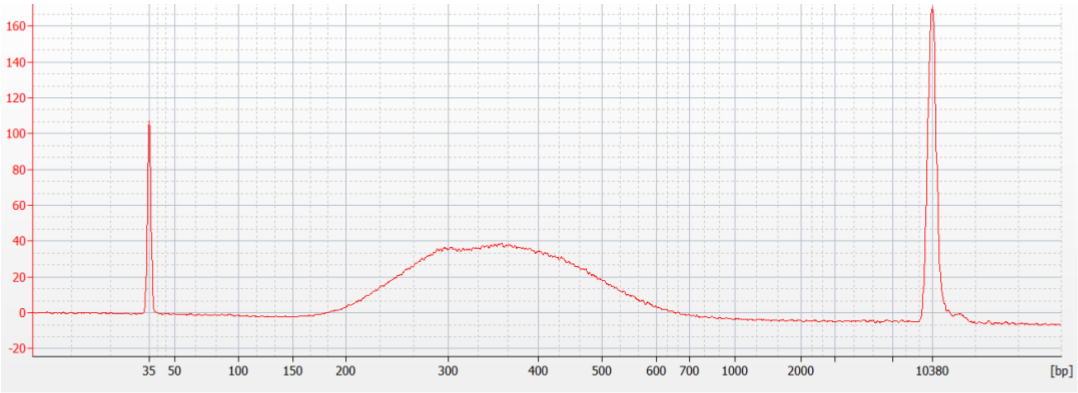

Figure 2: Example High Sensitivity Bioanalyzer trace of a ppmSeq™ library generated from 100 ng gDNA

2. Measure the library concentrations using the NEBNext® Library Quantification Kit for UG Libraries as described in the UG. Library Quantification Protocol (P00064).

9. Best Practices

The following recommendations are provided as a general guideline:

General Dilution Tips

1. Use freshly calibrated pipettes and certified free-from-DNAse/RNAse tips to prevent contamination and ensure accurate pipetting.
2. Minimize exposure to physical stress and light, especially for fluorescent dye-based methods, to prevent degradation and quenching.
3. Label all tubes clearly and keep a detailed log of dilutions to avoid sample mix-ups.
4. Work quickly but carefully to prevent evaporation, which can affect dilution factors, especially in small volumes.
5. Consider the impact of evaporation and adsorption in low-volume dilutions and use low-binding tubes and tips to minimize sample loss.

Dilution Guidelines and Factors for Library Preparation

Dilution Factors for Starting Samples:

DNA: For library preparation, an initial DNA concentration of 1-10 ng/µL is often required. If the starting DNA concentration is 50 ng/µL, a dilution of 1:5 or 1:10 may be appropriate to achieve this range.

General Dilution Guidelines:

1. Serial Dilutions: For samples with high concentrations, serial dilution is the preferred method to minimize error. A standard approach might be a 1:10 dilution followed by a 1:100 dilution, effectively resulting in a 1:1000 overall dilution.
2. Low-Volume Samples: When handling small amounts, it is advisable to avoid diluting less than 2 µL of the starting material or library into the final diluent volume, to prevent significant loss of volume during pipetting.

10. Safety and Regulatory

General

Using this product in a manner not specified in the user documentation may result in personal injury or damage to the instrument or device. Ensure that anyone using this product has received instructions in general safety practices for laboratories and the safety information provided in this document.

Wear appropriate personal protective equipment before working with reagents and operating the instrument.

Waste

Dispose waste in accordance with applicable regional, national, and local laws and regulations. Refer to kit SDS(s) for disposal considerations.

Safety Data Sheets (SDS)

Safety Data Sheets (SDS) for reagents described in this user guide can be found in the following link: https://regulatory.ultimagenomics.com/public

Contact support@ultimagen.com for further questions regarding reagent SDS's.

Protocol Use

RUO – For Research Use Only. Not for use in diagnostic procedures.
